# Selenium Interactions with Algae: Chemical Processes at Biological Uptake Sites, Bioaccumulation, and Intracellular Metabolism

**DOI:** 10.3390/plants9040528

**Published:** 2020-04-19

**Authors:** Dominic E. Ponton, Stephanie D. Graves, Claude Fortin, David Janz, Marc Amyot, Michela Schiavon

**Affiliations:** 1 GRIL, Département des Sciences Biologiques, Université de Montréal, Montreal, QC H2V 0B3, Canada; m.amyot@umontreal.ca; 2 Toxicology Graduate Program, University of Saskatchewan, Saskatoon, SK S7N 5B3, Canada; s.graves@usask.ca; 3 EcotoQ, Institut National de la Recherche Scientifique, Centre Eau, Terre et Environnement, QC G1K 9A9, Canada; claude.fortin@ete.inrs.ca; 4 Department of Veterinary Biomedical Sciences, University of Saskatchewan, Saskatoon, SK S7N 5B4, Canada; david.janz@usask.ca; 5 DAFNAE Department, Università degli Studi di Padova, 35020 Legnaro, Italy; michela.schiavon@unipd.it

**Keywords:** algae, selenium, competition, sulfate, partition coefficient, selenate, selenite, organic selenium

## Abstract

Selenium (Se) uptake by primary producers is the most variable and important step in determining Se concentrations at higher trophic levels in aquatic food webs. We gathered data available about the Se bioaccumulation at the base of aquatic food webs and analyzed its relationship with Se concentrations in water. This important dataset was separated into lotic and lentic systems to provide a reliable model to estimate Se in primary producers from aqueous exposure. We observed that lentic systems had higher organic selenium and selenite concentrations than in lotic systems and selenate concentrations were higher in lotic environments. Selenium uptake by algae is mostly driven by Se concentrations, speciation and competition with other anions, and is as well influenced by pH. Based on Se species uptake by algae in the laboratory, we proposed an accurate mechanistic model of competition between sulfate and inorganic Se species at algal uptake sites. Intracellular Se transformations and incorporation into selenoproteins as well as the mechanisms through which Se can induce toxicity in algae has also been reviewed. We provided a new tool for risk assessment strategies to better predict accumulation in primary consumers and consequently to higher trophic levels, and we identified some research needs that could fill knowledge gaps.

## 1. Introduction

In ecotoxicology, the essential micronutrient selenium (Se) has emerged as an aquatic pollutant of global concern [1]. This is due in part to the narrow range between essentiality and toxicity, particularly in yolk-bearing vertebrate animals such as fishes, aquatic birds, and amphibians [2]. Anthropogenic activities such as discharges of coal, uranium, phosphate and base metal mining effluents, coal-fired electricity generation wastes (i.e., coal combustion residuals), oil refining wastewaters, and agricultural irrigation of seleniferous soils can greatly increase the naturally occurring inputs of Se into aquatic systems and place susceptible aquatic species at ecotoxicological risk (see case studies reviewed in References [3] and [4]). These case studies documenting negative impacts of anthropogenic inputs of Se in aquatic systems on fish and bird populations provide some of the clearest cause–effect relationships in the history of ecotoxicology.

The speciation, biotransformation, and cycling of Se in aquatic systems are more complex than most trace elements, and dictate its fate and toxicological effects in aquatic ecosystems [5,6,7]. Although inorganic selenate (Se(VI)) and selenite (Se(IV)) are the dominant forms (species) of Se in surface waters, Se toxicity to aquatic wildlife such as fishes and birds is governed by the bioavailability of organic Se species in food webs [7,8]. In aquatic ecosystems contaminated with excess Se, microalgae play fundamental and critical roles in the ecotoxicological risk of Se to higher trophic level organisms because they (1) rapidly and efficiently take up dissolved inorganic forms of Se from the water column, (2) biotransform these inorganic Se species to organic selenide compounds (org-Se; Se(-II)) such as the seleno-amino acids selenocysteine (SeCys) and selenomethionine (SeMet), and (3) transfer these organoselenides by dietary pathways to primary consumers such as zooplankton, benthic invertebrates, and fishes that graze on periphyton [5,8,9,10]. Higher trophic level consumers then further bioaccumulate Se by feeding on these primary consumers. Thus, Se behaves more like mercury and persistent organic pollutants than other trace elements due to its ability to bioaccumulate, and indeed biomagnify, within aquatic food webs [7].

Unlike most trace elements, the speciation, bioaccumulation, and trophic transfer of Se are poorly predicted by thermodynamic or fugacity models, because biological (kinetic) processes are involved [7,11]. These kinetic processes are due to the essentiality of Se, where saturable transporters that recognize Se(VI) and Se(IV) have been reported in certain microalgae [12,13,14] and saturable transporters for SeMet have been reported in the gut of fish [15]. In addition, most organisms, including algae, are able to metabolically biotransform Se to a variety of biochemical intermediates in vivo [8,16]. Biodynamic modeling, which considers the combined influence of trace element concentration in water and such physiological processes [17], is the current approach used by many scientists and government agencies to enable accurate predictions of ecotoxicological hazard in risk assessments for Se [7,18]. The first step in a Se biodynamic model is termed the partition coefficient (*k_d_*; also referred to as enrichment factor) [19], which describes the ratio of Se concentration in particulates to the dissolved aqueous total Se concentration ([TSe]). Particulates are operationally defined as living (e.g., algae, periphyton, and bacteria) and non-living (e.g., organic material) components in the water column or at the sediment–water interface. Importantly, due to several environmental factors, *k_d_*s for Se can vary by several orders of magnitude [7,8,9]. In contrast to the wide range of *k_d_*s, trophic transfer factors (TTFs) for Se (the ratio of Se concentration in consumer to Se concentration in diet) usually vary only from 1 to 5 and are commonly about 2 [8]. The large variation in the partitioning at the base of food webs creates much uncertainty in ecological risk assessments for Se, and is the main reason why water quality guidelines to protect aquatic biota based on aqueous Se concentration are being changed to tissue-based water quality guidelines in certain jurisdictions [18,20]. Thus, a better understanding of factors influencing algal uptake of Se is an area with many data gaps and research opportunities to better understand Se biodynamics in ecotoxicology.

Due to the important role of algae in the biogeochemistry, cycling, and toxicity of Se in aquatic ecosystems, and the ecotoxicological significance of Se, the overall goal of this manuscript is to provide a critical review of studies that have reported algal Se enrichment and modifying factors such as Se concentrations, Se speciation, water chemistry, and algal community structure. Furthermore, we propose a conceptual model of intracellular Se metabolism and discuss the overall results within the context of ecological risk assessments of Se.

## 2. Data Analysis

### 2.1. Partition Coefficient

**Figure 1:**
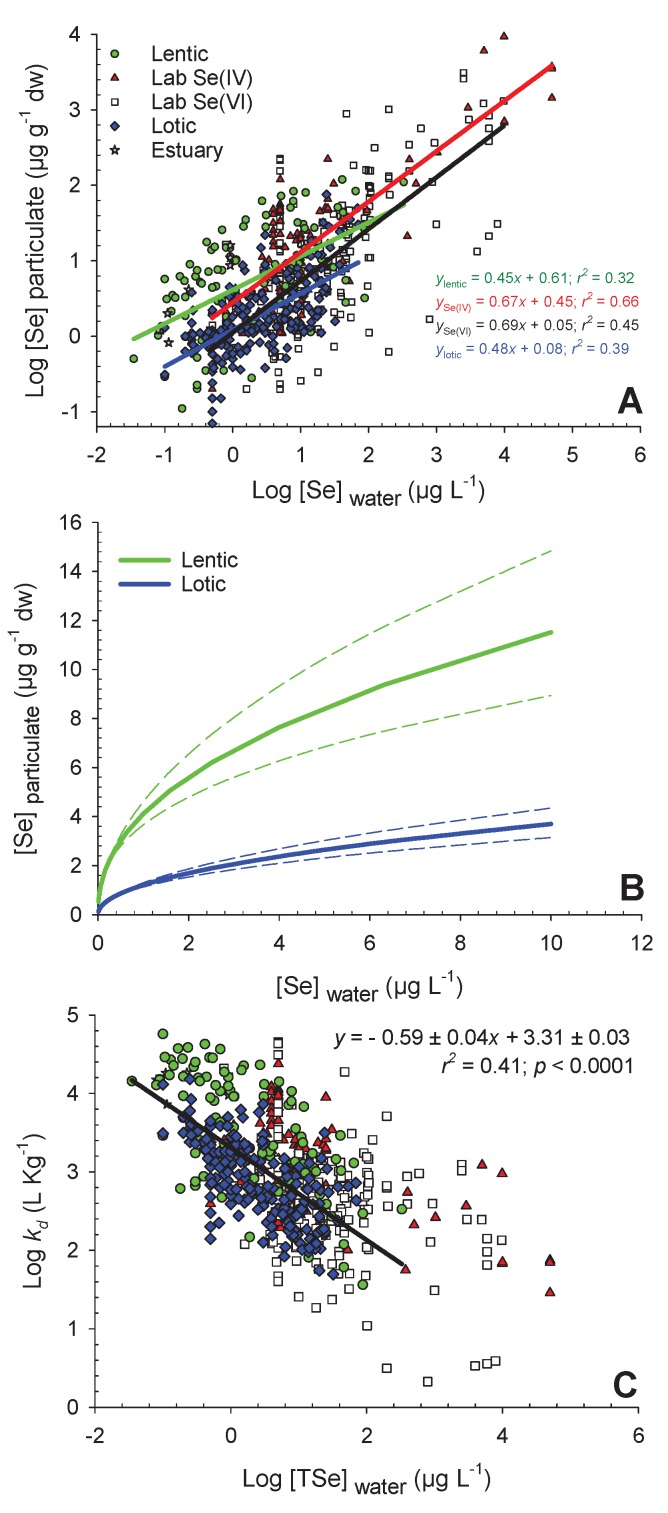
Panel (**A**) shows log-transformed selenium concentrations in particulate (Log [Se]_particulate_; µg g^−1^ dw) as a function of log-transformed water total dissolved selenium concentrations (Log [TSe]_water_; µg L^−1^) for lentic (green circles; *n* = 110), estuary (gray stars; *n* = 8), lotic (blue diamond; *n* = 195), selenate (white squares; *n* = 162), and selenite studies in the laboratory (red triangles; *n* = 94). Regressions are presented except for estuaries and equations are presented in panel **A** (*p*-value < 0.0001). Panel (**B**) shows the same regressions (± dashed standard errors) as in panel **A** for lentic and lotic systems but on a normal scale at relatively low aqueous Se concentrations (0-10 µg L^−1^). Panel (**C**) shows log-transformed partition coefficient (Log *k_d_*; L kg^−1^) as a function of log-transformed water total dissolved Se concentrations (Log [TSe]_water_; µg L^−1^) for the same systems as in panel **A** (see legend in panel **A**). The regression in panel **C** is for all field studies.

Data and references to produce Figure 1 can be found in the Supplementary Information (Table S1). These data were in part from the extensive reviews of Presser and Luoma [7] and DeForest et al. [19]. We also included data from laboratory-exposed algae to Se(VI) and Se(IV) used for the competition model (Section 2.3.). Data were log-transformed to respect normality and homoscedasticity. The regression slopes and intercepts for lotic and lentic systems were used at fictitious aqueous Se concentrations to draw the model in Figure 1B. Partition coefficients (*k_d_*) were calculated as:

(1) $$k_{d}=\frac{{\left[\mathrm{Se}\right]}_{\mathrm{particulate}}}{{\left[\mathrm{TSe}\right]}_{\mathrm{water}}}$$

where *k_d_* (L kg^−1^) is the Se concentration in particulates ([Se]_particulate_ (µg kg^−1^)) divided by the total dissolved water Se concentration ([TSe]_water_ (µg L^−1^)) from a given aquatic system. Particulates include algae, macroalgae, sediments, seston, periphyton, or microplankton from different aquatic ecosystems classified as lotic, lentic, or estuary. Lotic habitats include creeks and rivers. Lentic habitats include lakes, ponds, marshes, and salt lakes. Note that we do not use a capital “K” because *k_d_* is a constant of proportionality that varies and not an equilibrium constant.

### 2.2. Water Selenium Concentrations

Data and references were obtained from the extensive review of Conde and San Alaejos [21] (table not presented) and from Supplementary Table S1 (without laboratory studies). More studies were added and presented in Supplementary Table S2 with corresponding references. Water [Se] obtained from the literature were usually measured from 0.45 µm filtered water. Values below the detection limit (different among studies) were given a value -half of the detection limit. When a range of water [Se] was presented, we used the median value from this range. Data were log-transformed to respect normality.

For sites where [TSe] and speciation data were available, we calculated the proportion of Se species (Prop. Se species, %) by:

(2) $$\mathrm{Prop}.\;\mathrm{Se}\;\mathrm{species}=\frac{{\left[\mathrm{Se}\left(\mathrm{VI}\right)\right]}_{\mathrm{water}},{\left[\mathrm{org}-\mathrm{Se}\right]}_{\mathrm{water}},\;\mathrm{or}\;{\left[\mathrm{Se}\left(\mathrm{IV}\right)\right]}_{\mathrm{water}}}{{\left[\mathrm{TSe}\right]}_{\mathrm{water}}}\times 100$$

where at the numerator, there are either the selenite concentrations ([Se(IV)]_water_; µg L^−1^), the selenate concentrations ([Se(VI)]_water_, µg L^−1^), or the organic selenium concentrations ([org-Se]_water_; µg L^−1^). The latter includes all the operationally defined reduced Se forms [22]. At the denominator is the total dissolved Se concentrations ([TSe]_water_; µg L^−1^), including the three Se species.

### 2.3. Competition Model for Selenite and Selenate

Algae selenium bioaccumulation or uptake rate (12 h considered as steady state, [23]) were converted to concentrations in µg g^−1^ dry weight (dw) assuming that dry weight of the microalgal biomass makes 25% of fresh weight and density of fresh cells is 1.01 kg L^−1^ [24,25,26]. We also assumed that most cells have a spherical shape with an average diameter of 6.1 μm, thus cell volume was calculated as 119 μm^3^ [27].

Selenate and Se(IV) can interact with transport sites as described by the Biotic Ligand Model (BLM; [28,29]). Based on thermodynamic equilibrium reactions, Se(VI) and Se(IV) binding with a biotic ligand, ≡*Y*^+^, can be represented as follows (only Se(VI) shown):

(3) $$\equiv Y^{+}+SeO_{4}^{2-}\overset{K_{SeO_{4}}}{\Leftrightarrow }\equiv Y-SeO_{4}\overset{k_{int}}{\to }\equiv Y^{+}+{\left(SeO_{4}^{2-}\right)}_{cell}$$

(4) $$\left\{\equiv Y-SeO_{4}\right\}=K_{SeO_{4}}\cdot \left[SeO_{4}^{2-}\right]\cdot \left\{\equiv Y^{+}\right\}$$

(5) $$J_{int}=k_{int}\cdot K_{SeO_{4}}\cdot \left[SeO_{4}^{2-}\right]\cdot \left\{\equiv Y^{+}\right\}$$

where $K_{SeO_{4}}$ is the equilibrium constant for the reaction of Se(VI) with the biotic ligand ≡*Y*^+^, *k_int_* is the rate constant for Se internalization, and *J_int_* is the Se internalization flux. To simplify, charges on complexes were omitted. Curly brackets {} correspond to surface concentrations, square brackets [] to solution concentrations, and parentheses () to cellular concentrations.

Considering all possible surface complexation reactions, a membrane transport site (or biotic ligand) can bind either a Se(VI) ion (Equation (4)) or a competing anion such as sulfate (SO_4_^2−^; Equation (5)):

(6) $$\equiv Y^{+}+SeO_{4}^{2-}\overset{K_{SeO_{4}}}{\Leftrightarrow }\equiv Y-SeO_{4}\;K_{SeO_{4}}=\frac{\left\{\equiv Y-SeO_{4}\right\}}{\left\{\equiv Y^{+}\right\}\cdot \left[SeO_{4}^{2-}\right]}$$

(7) $$\equiv Y^{+}+SO_{4}^{2-}\overset{K_{SO_{4}}}{\Leftrightarrow }\equiv Y-SO_{4}\;K_{SO_{4}}=\frac{\left\{\equiv Y-SO_{4}\right\}}{\left\{\equiv Y^{+}\right\}\cdot \left[SO_{4}^{2-}\right]}$$

The total number of sites is given by the following mass balance equation:

(8) $${\left\{\equiv Y\right\}}_{T}=\left\{\equiv Y^{+}\right\}+\left\{\equiv Y-SeO_{4}\right\}+\left\{\equiv Y-SO_{4}\right\}$$

To express this concentration as a function of the concentration of free binding sites, we can combine Equations (6), (7), and (8):

(9) $${\left\{\equiv Y\right\}}_{T}=\left\{\equiv Y^{+}\right\}\cdot \left(1+K_{SeO_{4}}\cdot \left[SeO_{4}^{2-}\right]+K_{SO_{4}}\cdot \left[SO_{4}^{2-}\right]\right)$$

Equations (5) and (9) combined give:

(10) $$J_{int}=\frac{J_{max}\cdot K_{SeO_{4}}\cdot \left[SeO_{4}^{2-}\right]}{1+K_{SeO_{4}}\cdot \left[SeO_{4}^{2-}\right]+K_{SO_{4}}\cdot \left[SO_{4}^{2-}\right]}$$

where the maximum internalization rate *J_max_* = *k_int_ Y_T_*. By extension from the BLM, SO_4_^2−^ will inhibit Se(VI) uptake competitively: as the [SO_4_^2−^] increases, the product $K_{SO_{4}}\cdot \left[SO_{4}^{2-}\right]$ will also increase, leading to a decrease in *J_int_*.

Finally, the Se content at steady state can be described by the following equation:

(11) $${\left(SeO_{4}^{2-}\right)}_{cell}=\frac{J_{int}}{\mu }$$

where *µ* is the cellular growth rate (e.g., in divisions per hour). Combining Equations (10) and (11), and regrouping constant terms (*µ, J_max_* and $K_{SeO_{4}}$) gives:

(12) $${\left(SeO_{4}^{2-}\right)}_{cell}=F\frac{\left[SeO_{4}^{2-}\right]}{1+K_{SeO_{4}}\cdot \left[SeO_{4}^{2-}\right]+K_{SO_{4}}\cdot \left[SO_{4}^{2-}\right]}$$

where *F* is an adjustable parameter. The equation can be further simplified if the product of $K_{SO_{4}}\cdot \left[SO_{4}^{2-}\right]$ is >> 1. This is a reasonable assumption considering that SO_4_^2−^ is ubiquitous and often abundant in natural systems. In such a case, the unity term in the denominator can be omitted.

## 3. Global Selenium Exposure and Accumulation in Algae

### 3.1. Synthesis of Field Data

Algal Se accumulation varied over three orders of magnitude (from 0.10 to 120 µg g^−1^ dry weight (dw)) across the range of water [TSe] (0.035 to 330 µg L^−1^) from field studies (Figure 1A). Large differences in Se accumulation at a given aqueous [TSe] were observed (Figure 1A). Several factors, in addition to water [TSe], can contribute to the variability observed in algal [Se] across the studies reviewed. This section will provide a summary of the main (known) drivers of variability in Se accumulation at the base of the food web and will highlight knowledge gaps regarding the large variability from this global Se dataset. 

For all field data combined (Figure 1A), water [TSe] only explained 25% (*r^2^*) of the variability in algal [Se] (regression not presented). Regression from field data was separated according to lentic and lotic field studies (Figure 1A) and those explained 32% and 39% of the algae [Se] variability, respectively. Both regression slopes were similar (lentic: 0.45 ± 0.06; lotic: 0.48 ± 0.04; ± standard error (± SE)), but the *y*-intercept for lentic (0.61 ± 0.05) field studies was higher than for lotic ones (0.08 ± 0.03). These intercepts provide information on the overall difference in bioaccumulation potential between lentic and lotic systems. The difference in intercepts represents 3 ± 1 µg g^−1^ dw at 1 µg Se L^−1^ (10^0^ µg Se L^−1^) in water. The difference between systems has been shown on an untransformed scale in Figure 1B at relatively low aqueous [TSe], from 0 to 10 µg L^−1^. This model was performed from a large number of lotic (*n* = 195) and lentic (*n* = 110) sites and can therefore be used in Se risk assessment strategies to approximate [Se] in particulates from aqueous [TSe].

A very large *k_d_* range (3.5 orders of magnitude) was observed in field studies (from 37 to 57,000 L kg^−1^) with lower exposure concentrations generally associated with higher *k_d_*s (Figure 1C). The linear relationships between log-transformed particulate [Se] and water [Se] (Figure 1A) indicates a power relationship between the raw values (Figure 1B) that plateaus at high aqueous [TSe]. This is consistent with many laboratory studies that have reported that Se biological uptake sites become saturated at high aqueous [Se], and that Se accumulation can be fitted with Michaelis–Menten uptake curves [9,12,30]. Figure 1B shows the two Michaelis–Menten-type curves representing field data observations from Figure 1A at water [TSe] from 0 to 10 µg L^−1^.

The relationship of *k_d_* values as a function of water [TSe] (Figure 1C) may be useful in risk assessment strategies to better estimate *k_d_* depending on the contamination level. Note that there are two reasons for the negative relationship in Figure 1C, first the saturation of uptake sites on algae as presented in Figure 1A and Figure 1B and second, the presence of water [TSe] at the denominator in the formula to calculate *k_d_* (Equation (1)) that leads to a dependency between *k_d_* (*y*-axis) and water [Se] (*x*-axis). Even so, this relationship could be useful for Se risk assessment strategies since variation in *k_d_*s is one of the main factors that leads to variability in estimating [Se] up the food webs [7,19].

### 3.2. Lentic and Lotic Hydrology and Biogeochemistry

Several studies have assessed differences between lentic and lotic environments within a single watershed and have found that in general, higher Se accumulation is observed in lentic systems [31,32]. In the Elk Valley watershed (British Columbia, Canada), an area primarily composed of fast-flowing streams and rivers with occasional wetlands and oxbow lakes, [Se] in biota were greater in lentic compared to lotic habitats, and this was attributed to greater formation of org-Se and greater turnover of Se within lentic habitats [31]. In Benton Lake (Montana, USA), the [TSe] was greater in the inflow creek (62−560 µg L^−1^) compared to the wetland system (0.7−26 µg L^−1^), but the proportion of aqueous org-Se was higher in the wetlands/ponds (25%−59 % org-Se) than in the inflow creek (1%−25% org-Se) [33]. In the Jordan River drainage basin leading to the Great Salt Lake (Utah, USA), particulate [TSe] in lentic ponds was 2- to 5-fold higher than in lotic creek particulates [32].

The hydrology and biogeochemistry of an aquatic system influence the extent of Se bioaccumulation in algae. Residence time is a general indicator of several characteristics of a system that can influence Se uptake [34]. Waterbodies with low residence times (i.e., lotic systems) are characterized by high-flow and highly oxygenated conditions, whereas waters with high residence times (i.e., lentic systems) have low-flow and low-oxygen (reducing) conditions with greater biological activity [34]. Systems with higher residence times are typically correlated with greater biological turnover of Se, leading to higher particulate and algal Se relative to dissolved concentrations and greater food web assimilation of Se [35]. Receiving waters with high residence times are conducive to higher rates of dissimilatory reduction of Se, where algae and bacteria can reduce the oxidized, highly soluble form of Se (Se(VI)) to the more reduced and highly assimilable Se(IV), or even further to elemental (Se(0)) or org-Se (see Section 6) [36]. High residence times also increase the propensity for recycling biotic or abiotic Se from sediment back into solution in the form of Se(IV) or org-Se, leading to uptake of reduced Se forms by algae and assimilation into the aquatic food web. Due to the greater uptake rates of reduced and org-Se relative to the most oxidized form (Se(VI)), and the pronounced reducing conditions in slow-moving waters, Se bioaccumulation in biota tends to be higher in slow-moving waters compared to streams and fast-flowing rivers (Figure 1A,B) [31,32].

Biogeochemical parameters, such as sediment reduction, oxidation (redox) conditions, and the presence of organic matter, can also explain some of the differences in Se accumulation among sites. Sediment total organic matter is positively correlated with surface sediment Se concentrations and the percent organic matter is positively correlated with the adsorption of weak acids (like Se(IV)) at low pH [10,37]. In fast-flowing waters, fine organic sediments that are high in organic matter and originate from dead and decaying plant or animal matter are typically removed from the system quickly. In contrast, in slow-moving waters, detritus accumulates on bottom sediments providing an ideal substrate for microorganisms as periphytic biofilms to assimilate Se under reducing conditions. Those conditions are conducive to the recycling of Se from organic matter back into solution for uptake by algae [38]. Se-rich lentic pelagic organisms eventually die and deposit onto lake sediments, providing a rich source of org-Se for microorganisms, benthic invertebrates, and fish [39] that also participate in the org-Se turnover from sediment to the water column in lakes. 

### 3.3. Algae Selenium Bioaccumulation in Laboratory Studies

Laboratory studies conducted with Se(IV) and Se(VI) were separated to obtain the two regressions presented in Figure 1A. Both have similar slopes (Se(IV): 0.67 ± 0.05, Se(VI): 0.69 ± 0.06) but are higher than those from field studies (lentic: 0.45 ± 0.06, lotic: 0.48 ± 0.04). Higher slopes are probably a consequence of higher bioaccumulation potential of pure cultures in contrast to field microorganism communities (often including inorganic materials). Both laboratory-derived regressions have significantly different intercepts (Se(IV): 0.45 ± 0.08, Se(VI): 0.05 ± 0.10) similarly to what was observed for lentic and lotic field studies. The difference in intercepts represent 2 ± 1 µg g^−1^ dw on an untransformed scale at 1 µg L^−1^. This intercept difference between Se(IV) and Se(VI) uptake is similar to the one observed between lentic and lotic field studies (3 ± 1 µg g^−1^ dw). These results suggest that Se bioaccumulation in lentic aquatic systems seems predominantly related to uptake of reduced Se species, such as Se(IV) in contrast to the lower intercept for Se(VI) studies that correspond better with lotic system studies.

There was a difference in the range of water [TSe] used in laboratory and field studies (Figure 1A). The [TSe] from laboratory studies ranged from 0.5 to 50,000 µg L^−1^ while those from field studies ranged from 0.035 to 330 µg L^−1^. To increase the relevance of future laboratory studies, they should be conducted at water [Se] that reflect what is observed in natural systems. In particular, [Se] around 0.01 to 5 µg L^−1^ are relevant because they are in the range of the current United States Environmental Protection Agency (US EPA) and Canadian Environmental Quality Guidelines (CCME) water quality guidelines for the protection of aquatic life [18,40]. Given that the rate of Se accumulation generally decreases with increasing water exposure concentrations (Figure 1B), it is important to acknowledge that exposures at concentrations exceeding this range may not be useful for advancing our understanding of algal Se uptake kinetics under environmentally relevant conditions.

### 3.4. Taxonomic Differences in Se Accumulation

Taxonomic differences in Se accumulation among algal groups have been well-documented in the laboratory, and field-based studies have also illustrated differences in Se accumulation among food sources that can have significant impacts on Se exposure for higher trophic level organisms [9,41,42]. In monocultures of 14 different marine phytoplankton species, average *k_d_*s ranged from 4.2 × 10^1^ to 2.8 × 10^6^ and from 2.4 × 10^1^ to 1.5 × 10^5^ when exposed to 0.15 nM (0.01 µg L^−1^) and 4.5 nM (0.36 µg L^−1^) of Se(IV), respectively [9]. The main reasons proposed for the differences in uptake among species are differences in Se requirements and regulation (see Section 6).

Differences in Se accumulation among taxa have also been observed with field-collected organisms. Markwart et al. [42] used field-collected periphyton communities to determine community-driven differences in the uptake of Se(VI) and Se(IV), and found that Se accumulation differed among periphyton communities. Selenium accumulation was greater in communities dominated by cyanobacteria, and lowest in communities dominated by Bacillariophyta (i.e., diatoms). In order to determine the relative Se uptake by different components of biofilm in mine-affected streams in West Virginia (USA), Arnold et al. [43] applied density-fractionation techniques to separate filamentous green algae from diatoms and sediment fractions collected from the Mud River. In a mine-affected stream where composite biofilm [Se] was 2.7 µg g^−1^ dw, they found an 18-fold higher [Se] in the diatom/sediment fraction compared to the filamentous algae fraction. This study illustrates how a composite biofilm sample might be misleading in terms of the distribution of Se within the samples and predictions of Se transfer to the next trophic level. Ponton et al. [23] compared the uptake of different chemical Se species by *Chlamydomonas (C.) reinhardtii* and field-collected microplankton exposed under the same laboratory conditions. SeMet uptake by field-collected microplankton (surface water filtered on 64 µm and centrifuged) was 30-fold higher than for the green alga. They suggested that bacteria or the dominant taxa Dinophyceae and Cryptophyceae in the microplankton could be the reason for this large difference in uptake between taxa. Indeed, Dinophyceae and Cryptophyceae were also reported to have a high Se uptake by Baines and Fisher [9].

Among the field studies reviewed herein (Figure 1A), samples used to represent accumulation at the base of the food web were termed “particulates” and included sediment (including different depth sections), suspended particulate matter (sieved or not), seston, periphyton, macrophytes, filamentous algae, phytoplankton, detritus, macroalgae, or in some cases, a mixture of some of these components. To determine if some of the variability in particulate [Se] in the global dataset could be explained by the effect of different sample types, a factor was added to the regression analysis to separate algae, periphyton, and sediment, but this factor was not significant (*p* > 0.05) in predicting particulate [Se].

More research is needed to investigate the causes of differences in Se accumulation among complex natural communities such as algal communities, periphyton, and biofilm, since these differences in Se accumulation among taxa have implications for predicting Se trophic transfer to primary and secondary consumers. It is important to note that most microorganism communities in biofilms are composed of algae, fungi, bacteria, and detritus. Relative to algae, little is known about Se bioaccumulation in these other organisms, though they may be responsible for a significant portion of Se uptake in these complex communities [23,44].

Lower accumulation of Se in sediment relative to algae has been observed [31,45]. It is likely that the biotic components of measured particulate Se would be responsible for most of the accumulation, and the presence of inorganic materials may dilute the measured Se present. For instance, sediment percent organic matter (when reported) ranged from <1% to 90% among studies. In the future, standardizing the types of samples collected to represent the base of the food web will be important for accurately assessing algal Se accumulation and subsequent food web transfer. For example, pelagic communities should be sieved with a small-mesh-size synthetic nylon screening to remove zooplankton. Measuring additional parameters, like sediment depth and percent organic matter, and periphyton or particulate chlorophyll *a* and ash-free dry mass, may help to characterize the samples collected and would help to interpret the measured Se concentrations. Future studies could also be aimed at understanding the differences in accumulation between different taxa, for instance between bacteria, fungi, and algae.

### 3.5. Case Examples of High Selenium Bioaccumulation

**Figure 2:**
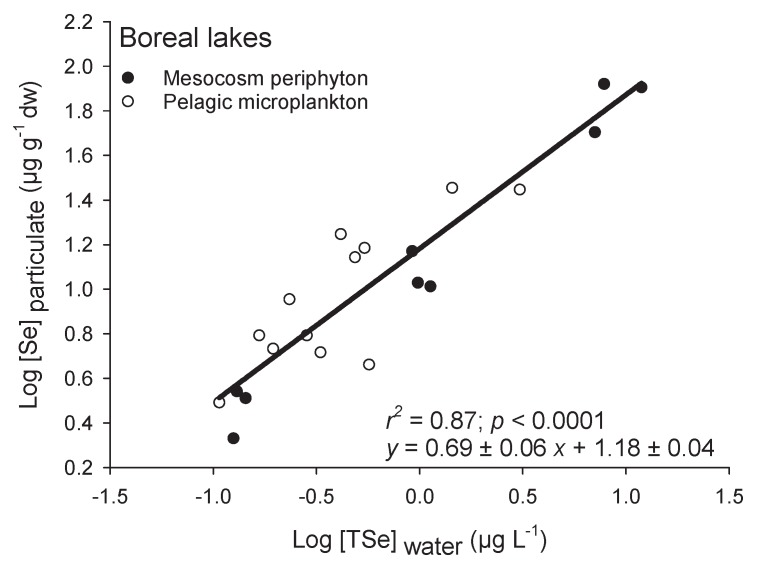
Log-transformed Se concentrations in particulate (Log [Se]_particulate_; µg g^−1^ dw) as a function of log-transformed water total dissolved Se concentrations (Log [TSe]_water_; µg L^−1^). Closed circles are from periphytic biofilms in mesoscosms spiked with Se(IV) in a boreal lake [45] and the open circles are from pelagic microplankton (<64 µm filtered and centrifuged water) collected in 12 boreal lakes in two mining regions [23]. Note that these are among the highest *k*_d_s reported in Figure 1C.

Regressions of algal Se accumulation as a function of water [TSe] in two separate studies (one field [23] and one mesocosm [45]) from boreal lakes in Ontario and Quebec (Canada) did not differ significantly in slopes or intercepts, suggesting that Se bioaccumulation across boreal lakes is similar (Figure 2). This is interesting because one study used in-lake enclosures to look at uptake of Se (added as Se(IV)) in periphyton and the other assessed microplankton (water filtered on 64 µm and centrifuged) Se accumulation in control and mine-affected lakes (org-Se from < 20% to more than 90%) [46]. The slope (0.69 ± 0.06; ±SE) and intercept (1.18 ± 0.04; ±SE) of the relationship between log-transformed algae and water [Se] for those two studies were almost twice those from overall field studies (Figure 1A), indicating a higher uptake of Se over exposure concentrations and an overall greater amount of Se being accumulated at a given exposure concentration. The intercept [Se] was 15 ± 1 µg g^−1^ at 1 µg L^−1^ in contrast to 4 ± 1 µg g^−1^ for the intercept of overall lentic habitats. This higher intercept for boreal lakes could be due to the speciation of Se, since in these studies, Se was present as Se(IV) and org-Se or added to enclosures as Se(IV). The [org-Se] in water was the best predictor of accumulation in the planktonic food chain from those boreal lakes [23,46]. Selenite, once added to the mesocosms, was likely readily reduced further by microorganisms and biotransformed to org-Se. Other potential reasons for the greater slopes and intercepts in boreal lakes include low nutrient (phosphate) and SO_4_^2−^ levels, and neutral or slightly alkaline pH [46,47,48,49]. Selenium accumulation among systems will be discussed below with the analysis of the prevalence of Se species in different water systems.

## 4. Aqueous Selenium Speciation and Concentrations in Aquatic Environments

Selenium and sulfur are two non-metal members of the chalcogen group, and consequently, non-metallic behavior governs Se geochemical cycling as well as biologically mediated reactions. Unlike metals, which typically exist as dissolved cations in water, Se is hydrolyzed in aqueous solution to form the oxyanions Se(VI) and Se(IV). These anions thus display increased solubility with increasing pH, in contrast to most metals where the opposite effect occurs.

The presence of different Se oxidation states from the reduced forms Se(-II, 0) to the oxidized forms Se(IV) and Se(VI) is primarily controlled by biogeochemical reactions. The most abundant Se species in natural waters are inorganic Se(IV) and Se(VI) and their relative abundance depend on the geological source of Se and the water physico-chemical properties (residence time, redox potential, and pH; [46,50,51]). The other primary Se species in water are org-Se [22], although they are usually thought to be at much lower concentrations than inorganic species. Reduced forms include the products of algal or microbial dissimilatory reduction of Se(VI) and Se(IV) (see Section 6). Selenium possesses one of the most complex speciation profiles of any trace element, with over 50 org-Se compounds identified to date [6]. Organic Se species in water include but are not limited to SeCys, SeMet, selenocyanate, dimethylselenide, selenoglutathione, and selenoneine [22,35,52], but very few studies have described those chemical species in natural waters. Bacteria and algae may also reduce inorganic Se to particulate elemental Se(0) as a means of detoxification [53]. Elemental Se is predominantly found in anoxic environments such as sediments, where chemical, physical, and biological factors promote its formation [6]. Elemental Se and org-Se were often analyzed together without distinction [22]. In this section, we will broadly review the current knowledge about Se concentrations and Se speciation in aquatic environments, and the factors leading to the prevalence of one form of Se over the others.

### 4.1. Total Selenium Concentrations

**Figure 3:**
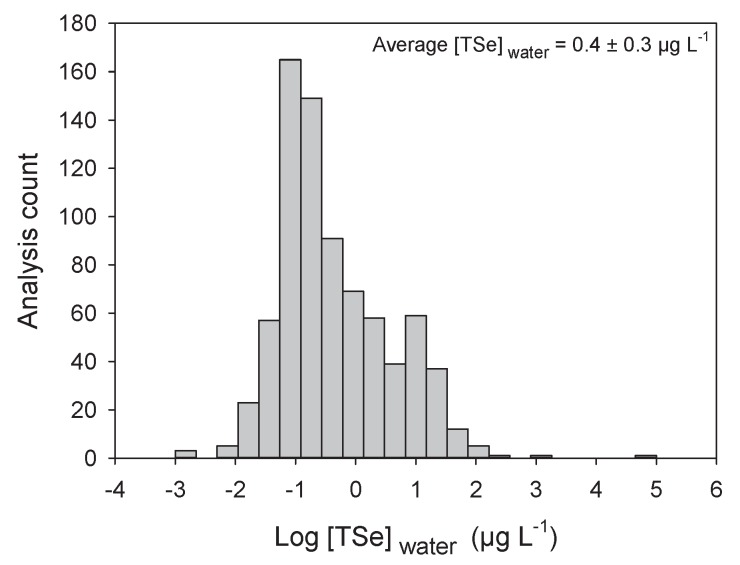
Frequency distribution of log-transformed total dissolved Se concentrations (Log [TSe]_water_; µg L^−1^) in water from around the world (*n* = 775). Average and standard error is presented in the graph.

**Figure 4:**
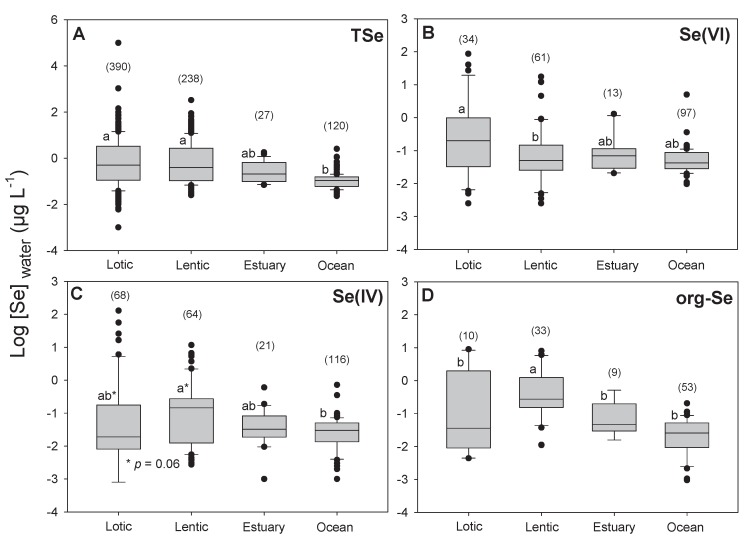
Boxplot of log-transformed (**A**) total dissolved selenium (TSe), (**B**) selenate (Se(VI)), (**C**) selenite (Se(IV)), and (**D**) organic selenium (org-Se) concentrations in water (µg L^−1^) for different aquatic systems categories (Lotic, Lentic, Estuary, Oceans (and seas)). Number of samples is presented in parentheses. Significant differences (analysis of variance (ANOVA) followed by Tukey’s test; *p* < 0.05) are presented by different lower-case letters.

The [TSe] in natural waters vary according to the surrounding geology. Earth crust Se concentrations vary from less than 0.1 to 0.5 µg g^−1^ in most igneous rocks to 1000 µg g^−1^ in the shales of the Phosphoria formation in central North America [54]. This high variation in soil Se content also leads to large variations in water [TSe] (Figure 3) [21,55]. Most uncontaminated aquatic systems tend to have [TSe] below 0.2 µg L^−1^ (Figure 3). The average [TSe] calculated from 775 samples from aquatic environments is 0.4 ± 0.3 (±SE) µg L^−1^ (Figure 3). As expected, [TSe] were significantly lower in seawaters than in freshwaters (*p* < 0.001; Figure 4A) but were similar among lotic, lentic, and estuary systems. Some of the highest [TSe] were encountered in aquatic systems receiving water from coal-mining operations [1] and from agricultural irrigation of semi-arid regions, such as, for example, California (330 µg L^−1^, [56]) and Pakistan (300–2000 µg L^−1^; [57]). The frequency distribution (Figure 3) presents fewer low concentrations than higher ones, probably because of the high detection limit of some instruments (≥0.2 µg L^−1^). Technologies such as atomic fluorescence spectrometry (AFS) or fluorometry provide adequate detection limits (close to 10 ng L^−1^) but these often require the transformation of Se species to Se(IV) prior to detection. Inductively coupled plasma-mass spectrometry (ICP-MS) provide relatively higher detection limits (≥0.1 µg L^−1^) but new systems such as ICP-MS-MS reduces interferences and allow detection in the same range as AFS. ICP-MS-MS coupled with liquid chromatography probably provides the best system currently available to detect Se species (see reviews [58] and [59] and example [52]).

### 4.2. Relative Abundance of Selenium Species

We found 775 data points for [TSe]. Speciation data were scarcer, we found 205 for Se(VI), 269 for Se(IV), and 105 for org-Se. The average concentrations for Se(VI), Se(IV), and org-Se were 0.07 ± 0.4, 0.04 ± 0.5, and 0.06 ± 0.3 µg L^−1^ (± SE), respectively. Overall, the proportion of Se(VI), Se(IV), and org-Se were 43% ± 27%, 31% ± 24%, and 36% ± 22%, respectively. These speciation results show for the first time that globally, Se(IV) and org-Se concentrations and proportions are similar. It is therefore very important to measure and characterize the different org-Se species in natural waters and their relative bioavailability.

Selenate concentrations were significantly higher in lotic than in lentic systems (*p* = 0.01; Figure 4B) and lentic systems had slightly higher [Se(IV)] than in lotic environments (*p* = 0.06; Figure 4C). Seawater had significantly lower [Se(IV)] than lentic systems (*p* = 0.03; Figure 4C). The [org-Se] were significantly higher in lentic systems than in the three other types of aquatic water bodies (Figure 4D). As mentioned previously, Se biogeochemical turnover in lakes is greater than in lotic systems, increasing the probability of reducing Se(VI) to Se(IV) and org-Se. Clearly, more information is needed about Se speciation and determination of the actual molecules included in operationally defined org-Se in water.

**Figure 5:**
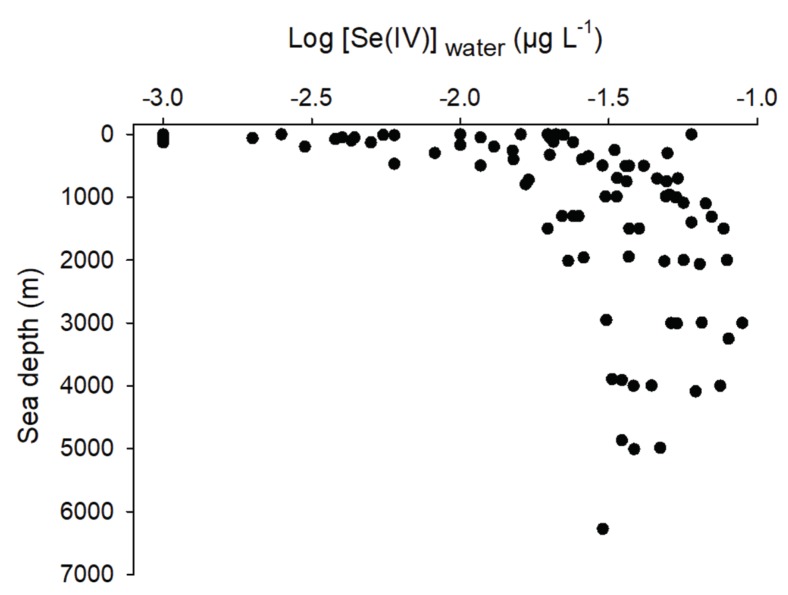
Log-transformed selenite concentrations (Log [Se(IV)]_water_, µg L^−1^) as a function of oceans depth (m) from several oceanic cruises (modified from Conde and Sanz Alaejos [21]).

Oxidizing conditions favor the presence of Se(VI) over other Se species [60]. For example, water originating from irrigation, runoff from shales, or downstream of coal mining activities with high pH generally have higher Se(VI) concentrations than those of Se(IV) [1]. Martin et al. [50] reported that oxidation-reduction potential was positively related to the proportion of Se(VI) in marshes downstream of coal mining activities as a function of depth. The opposite was also true for org-Se. Relationships between Se species concentrations as a function of depth were often observed in oceans. Deeper zones of lakes and oceans have higher concentrations of Se(IV). The extensive review of Conde and Sanz Alajeos [21] allowed us to look at the relationship of Se(IV) concentrations as a function of depth from several oceanic missions (Figure 5). Higher Se(IV) concentrations were measured in deeper water as opposed to org-Se. This phenomenon was explained by the absorption of Se(IV) and Se(VI) in shallow waters, reduction in cells to org-Se, and physiological excretion (org-Se peaks in surface waters) or release of org-Se as cells die in deeper waters. It was suggested that org-Se oxidation to Se(IV) is relatively fast but that further oxidation to Se(VI) is unlikely given the thermodynamic stability of Se(IV) in natural waters [61]. Thus, the similar Se(VI) and Se(IV) concentrations in deep waters observed in the latter study was a consequence of external sources (coastal waters) of Se(VI) and not from a regeneration process. This explained in part why we did not observe as clear of a relationship for [Se(VI)] as a function of depth as the one observed in Figure 5 with Se(IV). Retention time of a freshwater system has a similar effect on the distribution of Se species. In several ponds placed in series downstream of a Se(VI)-contaminated influent, Gao et al. [51] observed a build-up of reduced Se species (Se(IV) and org-Se) going from upstream to downstream of these ponds.

Higher pH and OH^−^ concentrations are positively related to higher desorption of Se anions from particulate material [62]. Positive relationships between inorganic [Se] and pH have often been observed [1,46,57,63]. Ponton and Hare [46] showed that for a range of pH among boreal lakes, the proportion of org-Se in water tended to decrease with increasing pH, in contrast to that of Se(IV). These two species were the most abundant (20% to 90% depending on pH) in contrast with low Se(VI) concentrations and proportions (21% ± 21%). Thus, the decline in the org-Se proportions as a function of pH was mostly a consequence of the [Se(IV)] rise with pH. Given this Se(IV) behavior as a function of pH, it is important to further our understanding of Se(IV) uptake by algae at different pH (see Section 5.2). 

**Figure 6:**
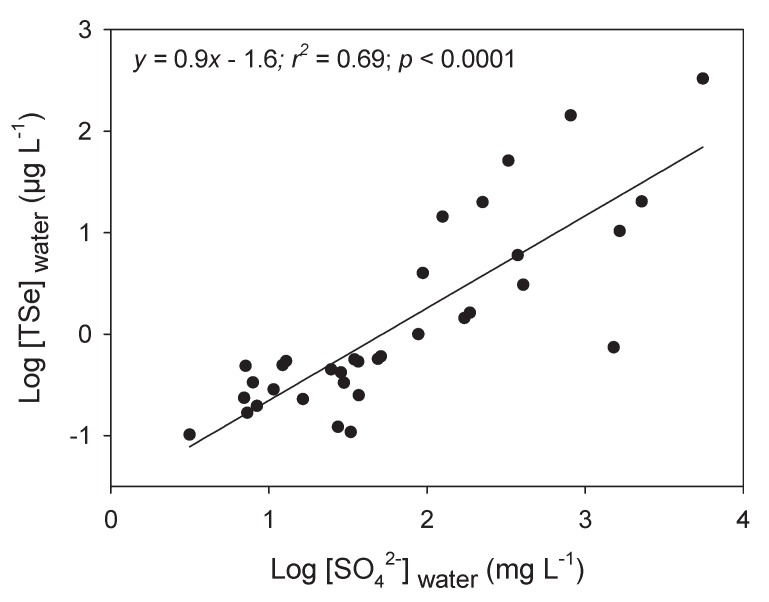
Log-transformed total dissolved Se concentrations (Log [TSe]_water_; µg L^−1^) as a function of log-transformed sulfate concentrations (Log [SO_4_^2−^]_water_; mg L^−1^) for boreal lakes in mining regions (Ponton and Hare [46]), coal operations-contaminated sites [50,65,66], and in Kesterson Reservoir [56].

Sulfur shares many similarities with selenium and both elements are present together in many systems, from pyrite to biological systems sharing similar cellular pathways [64]. In Figure 6, we present the relationship between SO_4_^2−^ and [TSe] in freshwaters to acknowledge their close relationship in aquatic systems. Unfortunately, we found few studies with both measurements. Seawater ([SO_4_^2−^ ] > 2500 mg L^−1^) or water from estuarine systems were not included because of their stable and high SO_4_^2−^ concentrations. Given the high interactions of both elements, studies on Se in the field and the lab should always measure and/or report [SO_4_^2−^] for risk assessment purposes and to better explain accumulation results (see Section 5.4 and Section 5.6).

Both aqueous Se speciation and bioaccumulation was rarely reported in the same field studies, thus it was not possible to directly explain the variability in algal Se accumulation (Figure 1A) by differences in Se speciation. Separating laboratory studies into the inorganic Se forms added and by lentic and lotic habitats suggested that Se(IV) and probably org-Se were the main forms being assimilated in lentic systems in contrast to Se(VI) in lotic systems. Though Se speciation is rarely measured or reported in bioaccumulation-related field studies due to both high costs and difficulty in analysis, it is extremely important in determining the extent of Se bioaccumulation in biota.

## 5. Water Chemistry and Selenium Bioavailability

Cationic metal uptake is generally known to depend strongly on water chemistry. The BLM was developed to incorporate the competing effects between cations and (cationic) metals [29,67]. The case of Se is more complex due to the diversity in (in)organic and redox forms. These forms are not assimilated at the same rate and are transformed once assimilated.

### 5.1. Uptake of Selenium Species

Organic Se has the highest uptake rates in algae compared to Se(VI) and Se(IV) (up to 3 orders of magnitude in Bailey et al. [68]). Fournier et al. reported a 3-fold higher uptake rate of SeMet compared to Se(IV) and Se(VI) in the presence of 100 µM SO_4_^2−^ by *C. reinhardtii*. A similar experiment with the same alga exposed to 5 µg L^−1^ of each Se species (100 µM SO_4_^2−^) led to similar results [23]. Interestingly, this last study showed that SeMet uptake was 30-fold higher for field microplankton exposed to the same laboratory conditions as the alga. The microplankton laboratory exposure allowed Ponton et al. [23] to adequately estimate field microplankton [Se] from several lakes. The *k_d_* for SeMet was two-fold greater than that of Se(IV) from low µg L^−1^ to 5 µg L^−1^, and both Se species were not affected by [SO_4_^2−^].

Several studies have directly compared the uptake rates of different forms of Se for algae and have found that organic forms of Se tend to be taken up more readily and to a higher extent than inorganic forms [23,35,69,70]. Earlier field and laboratory studies reported that Se(VI) uptake by algae or periphyton is generally lower (*k_d_*s range from 140 to 490) than for Se(IV) (*k_d_*s from 720 to 2800) and org-Se (*k_d_*s from 12,200 to 36,300) [7]. More recent studies have shown a similar trend for the Se oxyanions. Conley et al. [71] observed higher initial uptake of Se(IV) compared to Se(VI) by natural periphyton communities. In that study, they also observed a reduction of Se(VI) by the periphyton assemblage (potentially Se(VI)-reducing bacteria) to Se(IV) over time, and an uptake rate increase in relation to the presence of Se(IV), so that at the end of the experiment, there were no differences in uptake between the two species. While there is general agreement that org-Se is taken up most readily by algal species, there is some disagreement about which inorganic form (Se(VI) or Se(IV)) is taken up more readily. Some studies, in contrast to those described above, have observed greater uptake of Se(VI) in multiple species of green algae, suggesting that uptake rates for the two oxyanions could be taxon-specific [12,35]. Some of the differences in uptake rates observed could be due to inhibition by other ions from the culture media. For instance, SO_4_^2−^ and phosphate (PO_4_^3−^), which are typically added to culture media, can inhibit the uptake of Se(IV) and/or Se(VI) and as such, may change the conclusions about which oxyanion is taken up to a higher extent in a particular study.

In the next sections, we focus on the impacts of ionic composition of surface waters on the assimilation of Se species. In a similar manner to the BLM for cationic metals, we can anticipate that different negatively charged Se forms will be influenced by other anions in solution. These include mainly OH^−^, PO_4_^3−^, SO_4_^2−^, and CO_3_^2−^. In the next sections, we examine the potential mitigating role of anions and of the selenium speciation.

### 5.2. Role of pH

In addition to direct competition with other ions, other water chemistry parameters can influence the bioavailability and uptake of Se. The role of pH is complex however since it involves changes in both OH^−^ and CO_3_^2−^ (due to equilibrium with atmospheric CO_2_) anions but can also lead to acid-base reactions of the Se anion. Because the relative importance of each cannot be teased apart from one another in most studies, we will thus consider pH as a single parameter.

Selenate appears to be unaffected by pH and is consistently present in the deprotonated form (SeO_4_^2−^) across pH values observed in natural waters [72]. Results from the literature strongly suggest that Se(VI) uptake and/or toxicity is unaffected by pH within an environmentally realistic range (pH 5–9). Indeed, such tests using two green algae (*Chlamydomonas reinhardtii*, *Scenedesmus pannonicus* subsp. Berlin) and one fungus (*Aureobasidium pullulans*) showed little influence of pH [23,69,73]. 

In contrast to Se(VI), the acid-base reactions of Se(IV) are important at natural pHs. The deprotonated form of Se(IV) (SeO_3_^2−^) is present at higher pH, while the protonated form (HSeO_3_^−^) is predominant at lower pH [72]. There is no consensus on the impact of pH (up to 9) on Se(IV) uptake/toxicity. In three studies using the green alga *C. reinhardtii*, two found no clear influence of pH [47,69], while another indicated a strong effect [23]. In the latter study, algae Se uptake was highly correlated with the calculated SeO_3_^2−^ concentrations (*r^2^* > 0.99) and, it was suggested that the impact of pH would be related to the inorganic speciation of Se(IV). With a pK_a_ of 8.4, Se(IV) is mostly present in the fully deprotonated form above pH 8.4. Below this pH, HSeO_3_^−^ predominates, which would have a reduced electrostatic attraction for the membrane transporters compared to the divalent form. The role of pH on Se(IV) uptake and toxicity remains to be confirmed. The difference in results between the three studies may indicate that the specific exposure conditions (boreal lake reconstituted water in Ponton et al. [23]) may have obscured the effect of pH on Se(IV) uptake in the two other studies [47,69]. Only one study examined the influence of pH on the uptake of SeMet [23]. In this particular case, a three-fold increase in Se uptake was observed from pH 7.0 to 9.0. These results together suggest that the high prevalence of Se at high pH and the increase in uptake of Se(IV) and org-Se with pH would result in greater risk of Se impacts at high pHs, as observed by Wang et al. [74].

### 5.3. Role of Phosphate

We found no studies in which the addition of PO_4_^3−^ affected Se(VI) uptake or toxicity. On the other hand, Se(IV) uptake can be inhibited by PO_4_^3−^ [47,49,69,75]. Increasing [PO_4_^3−^] from 4.3 to 43 mg PO_4_ L^−1^ reduced Se(IV) uptake by 50% in a green alga [49]. Selenite partition coefficient in two marine diatoms (*Thalassiosira pseudonana* and *Skeletonema costatum*) and a green alga (*Chlorella autotrophica*) was reduced by 2.4- to 8.1-fold after increasing phosphorus concentrations from 0.05 to 22 mg P L^−1^ [75], while Morlon et al. [47] found no clear observable effect.

The actual relevance of PO_4_^3−^ inhibition of Se uptake is questionable. In natural waters, concentrations of total phosphorous range from less than 1 µg P L^−1^ in ultra-oligotrophic systems to >200 µg P L^−1^ in eutrophic conditions, and concentrations are typically between 10 and 50 µg P L^−1^ in uncontaminated freshwaters [34,40]. In general, the [P] required to inhibit Se uptake are close to 100 µg P L^−1^. The overall role of PO_4_^3−^ in regulating Se uptake in natural systems is thus unlikely to be very important as the ambient concentrations of P are usually very low, except for highly eutrophic systems. Based on the P concentrations used in the above studies, only hyper-eutrophic systems may potentially observe an inhibition of Se(IV) uptake by PO_4_^3−^.

### 5.4. Role of Sulfate

Sulfate is the anionic competitor of Se uptake that was most abundantly studied. The literature is unanimous with regards to the competitive effect of SO_4_^2−^ on Se(VI) uptake and toxicity to aquatic plants and algae. These studies comprise results of SO_4_^2−^-protective effects on Se(VI) bioaccumulation in: the duckweed *Lemna minor* and the green alga *Pseudokirchneriella subcapitata* [76], the green alga *Selenastrum capricornutum* [77], the aquatic macrophyte *Ruppia maritima* [68], the green alga *Chlamydomonas reinhardtii* [23,48,49,69], and in natural microplankton [23]. Moreover, Simmons and Emery [78] showed that phytochelatin synthesis by *Chlorella vulgaris* due to Se(VI) exposure decreased with the addition of SO_4_^2−^. 

The impact of SO_4_^2−^ on Se(IV) uptake and/or toxicity to algae is not as clear as for Se(VI). Indeed, several authors found significant but no notable effect of SO_4_^2−^ [23,47,49,68,69]. As for SeMet, results show that SO_4_^2−^ does not influence its uptake by the green alga *C. reinhardtii* [23] and by the aquatic macrophyte *Ruppia maritima* [68]. Table 1 reports the overall effect of SO_4_^2−^ and PO_4_^3−^ ions, as well as for pH on the uptake of different Se species by algae.

### 5.5. Other Ions

There is evidence that nitrate could reduce Se(IV) uptake rates by *C. reinhardtii* [47] and that silicate ions could decrease Se(IV) uptake by marine diatoms (*Thalassiosira pseudonana* and *Skeletonema costatum*) and a green alga (*Chlorella autotrophica)* [75]. It is hypothesized that Se(IV) can be taken up through a silicon transporter in algae [13]. In monocultures of *C. reinhardtii* amended from 10 to 200 µM Na_2_SiO_3_ and two [Se(IV)] (3 and 10 µg L^−1^), no effect on Se(IV) accumulation was observed [69]. However, the environmental pertinence of these competitive reactions in natural freshwater systems remains to be demonstrated.

### 5.6. Modelling Competition with Sulfate

Sulfate inhibition occurs at concentrations that are environmentally realistic and can have important implications for Se bioaccumulation and interpretation of the prevalence of inorganic Se species uptake. Some of the variability in algal Se accumulation in the global dataset presented in Figure 1A may be due to differences in Se speciation and to competition with SO_4_^2^^−^. Higher slopes for some systems may be due in part to a reduced competition with SO_4_^2^^−^, since [SO_4_^2^^−^] ranged across study systems from <1 to >5000 mg L^−1^ (Figure 6). Areas with high [SO_4_^2^^−^], for example, some mine-affected areas or seawaters, may result in Se uptake inhibition, leading to significantly lower algal Se accumulation at a given water [Se].

**Figure 7:**
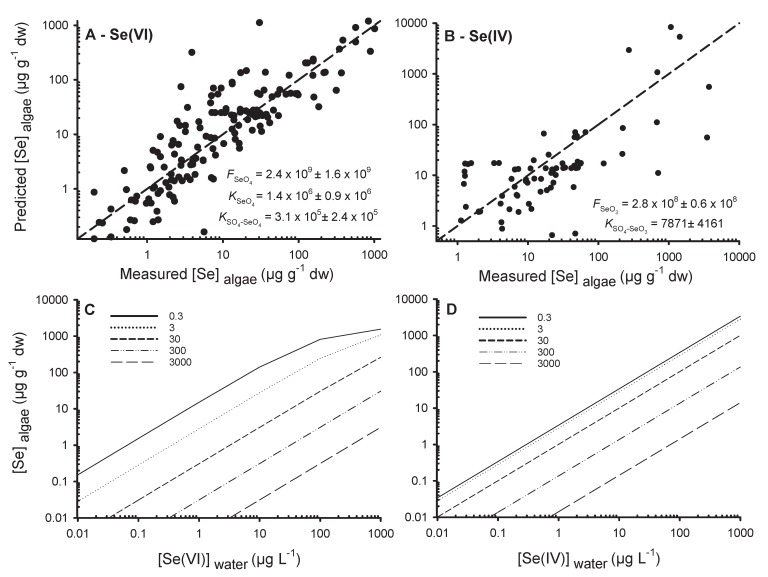
Predicted algae selenium concentrations (Predicted [Se]_algae_, µg g^−1^ dw) from the competition model (Equation (12)) of sulfate with (**A**) selenate (Se(VI)) and (**B**) selenite (Se(IV)) as a function of measured algae selenium concentrations (Measured [Se]_algae_, µg g^−1^ dw). The 1:1 lines and estimated constants (±standard errors) of the model (Equation (12)) have been shown. In (**C**) are the predicted [Se]_algae_ (µg g^−1^ dw, Equation (12)) as a function of fictitious selenate concentrations ([Se(VI)]_water_, µg L^−1^), (**D**) selenite concentrations ([Se(IV)]_water_, µg L^−1^) and sulfate concentrations (mg L^−1^; in legend). Note that to use both models with the constants provided, SO_4_^2−^, Se(IV) and Se(VI) concentrations must be in mol L^−1^ and the output results are in µg g^−1^ dw.

Data thus show that SO_4_^2−^ is likely to strongly modulate Se(VI) uptake in natural systems to a greater extent than Se(IV), while no competition effect has been noticed for SeMet [23,68]. We thus developed a model (Equation (12), Section 2.3) to predict Se(VI) and Se(IV) uptake as a function of ambient [SO_4_^2−^] using published data. Using this model, we fitted experimental data using non-linear regression and estimated parameters $F_{SeO_{4}}$, $K_{SeO_{4}}$, and $K_{{\mathrm{SO}}_{4}-{\mathrm{SeO}}_{4}}$, for Se(VI) exposures and $F_{SeO_{3}}$, $K_{SeO_{3}}$, and $K_{{\mathrm{SO}}_{4}-{\mathrm{SeO}}_{3}}$ for Se(IV) exposures (Figure 7). The selected data came from 16 and 13 studies for Se(VI) (*n* = 162) and Se(IV) (*n* = 94) exposures, respectively (Supplementary Table S3). 

Our model can adequately predict Se(VI) accumulation in algae using published data (Figure 7A). Studies encompassed four orders of magnitude in algae [Se]. The coefficient of determination (*r^2^*) was 0.17 for the simple regression analysis of algae [Se] as a function of [Se(VI)] (Figure 1A) and 0.78 comparing predicted and measured algae [Se]. The constants $F_{SeO_{4}}$, $K_{SeO_{4}}$, and $K_{{\mathrm{SO}}_{4}-{\mathrm{SeO}}_{4}}$, are presented in Figure 7A. In Figure 7C, the outputs of the model for different scenarios with a range of [Se(VI)] and [SO_4_^2−^] from oligotrophic lakes (0.3 mg L^−1^) to seawater (3000 mg L^−1^) are presented.

Competition model predictions for Se(IV) were not as consistent as for Se(VI) (Figure 7B). The simple regression of algae [Se] as a function of aqueous [Se(IV)] (Figure 1A) had a better *r*^2^ (0.66; Figure 1A) than that of the relationship of predicted as a function of measured algae [Se] (*r*^2^ = 0.59; Figure 7B). Albeit, the model is informative for comparison of Se(IV) and Se(VI) uptake physiology (Figure 7C,D). It was not possible to estimate $K_{SeO_{3}}$ because of the absence of a plateau for uptake studies with Se(IV), suggesting that Se(IV) uptake sites were not saturated at the highest aqueous [Se(IV)]. These high exposure concentrations had a strong influence on the sum of least squares for the determination of the constants $K_{SeO_{4}}$ and $K_{{\mathrm{SO}}_{4}-{\mathrm{SeO}}_{4}}$ and were leading to a global underestimation of the model compared to measured values. Consequently, we did not use the experiments where accumulation measurements were from 200 to 10,000 µg g^−1^ dw to estimate these constants. These results are nevertheless presented in Figure 7B (*x*-axis) and are in agreement with the modeled values. 

The estimated constant $K_{{\mathrm{SO}}_{4}-{\mathrm{SeO}}_{4}}$ was 7.9 × 10^3^, which is 39-fold lower than the constant determined for Se(VI) (3.1 × 10^5^), indicating the lower affinity of SO_4_^2−^ for binding on Se(IV) transporters than for Se(VI) transporters. Figure 7D presents the output of the Se(IV) model for the same range as for Se(VI) and [SO_4_^2−^] (Figure 7C). Our results suggest that Se(VI) uptake is greater than for Se(IV) at very low [SO_4_^2−^]. Uptake of Se(IV) is less impacted by SO_4_^2−^ than for Se(VI). Figure 7C,D can be used in risk assessment strategies to estimate algae [Se] at steady state and further to estimate [Se] in consumers using trophic transfer factors. For the first time, we provided a competition model on a large scale of Se concentrations including different algae species. This model allows us to better understand the inadequacy of results for uptake of inorganic Se. Uptake of Se(IV) has often been reported to be more important than uptake of Se(VI), however most studies were conducted at moderate or high [SO_4_^2−^]. At low [SO_4_^2−^], Se(VI) uptake can be more important than that of Se(IV).

## 6. Intracellular Selenium Metabolism

Once Se is taken up into algal cells, depending on interactions with all the possible environmental and chemical factors previously illustrated in this review, it is further subjected to assimilation processes. Therefore, in the next sections, several aspects of Se metabolism in algae will be thoroughly described.

### 6.1. Evolution of Selenium Metabolism 

In photosynthetic organisms, Se and sulfur (S) share common cellular uptake systems and metabolic routes [13,14,79,80]. Therefore, the biochemical aspects of Se/S assimilation in algae are expected to be similar to those described for plants, and the key enzymes involved in the chemical reactions are encoded by genes that display high similarity in sequence to their orthologous genes from plants [81,82]. However, one major difference is that those (micro)algae with a metabolic requirement for Se also possess molecular mechanisms for specific incorporation of Se-amino acids in essential selenoproteins (Sel or Sep) [83]. Such mechanisms were perhaps originally owned by plants as well, but then lost during evolution because of still unclear events [84]. It has been proposed that the plants’ habitat has played a role in this respect. Persistent availability of Se in the aquatic environment in particular, likely supported the need of Se for selenoproteins, which are functionally similar to thiol-based oxidoreductases [85,86]. Conversely, plants in terrestrial habitats were present in environments with sometimes low available Se concentrations and likely lost selenoproteins, replacing them with cysteine-containing homologs [84]. Higher oxygen concentration in the air may have contributed to this process by making highly reactive selenoproteins more vulnerable to oxidation processes in terrestrial plants, and thus promoting selection against the use of these proteins [87].

### 6.2. Selenate and Selenite Reduction and Assimilation in Cells

**Figure 8:**
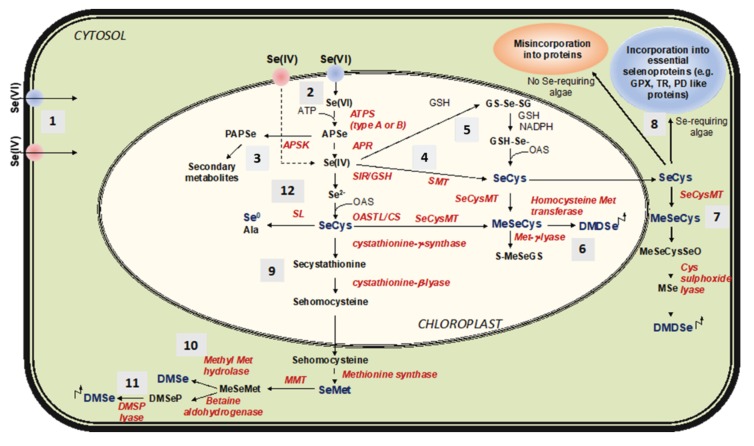
Schematic representation of the metabolic fates of Se in phototrophic organisms (plants and algae). (1) Selenate (Se(VI)) and selenite (Se(IV)) are the main forms of Se in aquatic environment taken up by algae. Once they have entered the cytosol, both ions are transported to the chloroplasts to be incorporated into selenocysteine (SeCys). (2) SeCys can be produced from Se(VI) through the sulfur assimilation pathway, which involves the enzymes Adenosine Triphosphate (ATP) Sulfurylase (ATPS), Adenosine Phosphosulfate Reductase (APR), and Sulfite Reductase (SiR). (3) Activated Se(VI) (Adenosine Phosphoselenate, APSe) can be phosphorylated by Adenosine Phosphosulfate (APS) Kinase (APSK) to produce Phosphoadenosine Phosphoselenate (PAPSe), which is a precursor of secondary Se-containing metabolites. (4) Se(IV) can be converted to SeCys directly by Selenomethyl Transferase (SMT). (5) Alternative pathway of Se(IV) reduction and SeCys synthesis involves glutathione (GSH), with selenodiglutathione (GS-Se-SG) produced as an intermediate. (6) SeCys can be methylated by Selenocysteine Methyl Transferase (SeCysMT) to form Methyl Seleno Cysteine (MeSeCys), which can be further converted to Dimethyl Diselenenide (DMDSe) through the action of Homocysteine Methyl transferase. SeCys can be transported outside the chloroplast and be either (7) methylated to produce MetSeCys and DMDSe or (8) incorporated into proteins (via a specific mechanisms in Se-requiring microalgae). SeCys in the chloroplast can be converted to Selenohomocysteine, which is then transformed into Selenomethionine (SeMet) once delivered to the cytosol. (9) SeMet can be methylated to produce Methylselenomethionine (MeSeMet), which is further converted to Dimethyl Selenide (DMSe) by either (10) methyl methionine hydrolase or (11) Dimethylselenopropionate lyase (DMSP lyase). SeCys can also be broken into Alanine (Ala) and elemental Se (Se^0^) by the activity of Selenocysteine lyase (SL). OAS: O-acetyl serine, OASTL/CS: OAS Thiol lyase/ Cysteine synthase, S-MeSeGS: S-Methyl-selenoglutathione, ϒECS: γGlutamyl Cysteine Synthetase, GluMeSeCys: Glutamylmethylselenocysteine, MMT: Methionine Methyl Transferase, DMSP: Dimethylselenopropionate, MeSeCysSeO: Methylselenocysteine Selenooxide, MSe: Methaneseleniol.

All the described possible metabolic fates of Se in phototrophic organisms, including algae, are depicted in Figure 8. After being absorbed by algal cells, Se moves to the chloroplasts and accesses the S metabolic pathway to be converted to selenide (Se^2−^), the ultimate substrate for the synthesis of SeCys and SeMet [14,80]. When Se(VI) is the main form of Se available for the uptake in aquatic environments, it is first reduced to Se(IV) in a rate limiting step that requests the sequential actions of two enzymes, adenosine triphosphate (ATP) sulfurylase (ATPS) and adenosine phosphosulfate reductase (APR) [13]. Both ATPS and APR are pivotal regulatory enzymes in S assimilation and, while isoforms with different subcellular localization (cytosolic or plastidial) exist in algae and plants, Se/S assimilation primarily takes place in the chloroplasts [80,88,89]. ATPS mediates the activation of Se(VI) by binding it to ATP to form adenosine phosphoselenate (APSe) [90]. Oceanic cyanobacteria and most eukaryotic algae possess an ATPS type-B containing seven to ten cysteines, while freshwater cyanobacteria have an ATPS type-A, with four conserved cysteine residues [89]. These two types of ATPS show different redox regulation capacity. APSe is further reduced to Se(IV) via transfer of two electrons from glutathione in a reaction catalyzed by APR [14,80,91], whose activity in some algae has been reported to be much higher (up to 400-fold) than in plants [81]. These first steps of Se/S assimilation in algae and plants are skipped whether Se(IV) is the major or preferred substrate for the uptake. Reduction of Se(IV) to Se^2−^ is then realized by both enzymatic and non-enzymatic reactions. In the first case, Se(IV) is reduced by the activity of sulfite reductase [14,80]. Otherwise, Se(IV) is reduced in a non-enzymatic two step-reaction where it is first converted to selenodiglutathione (GS-Se-SG) in the presence of GSH. GS-Se-SG is then reduced to selenopersulfide/glutathionylselenol (GS-SeH) by using reduced nicotinamide adenine dinucleotide phosphate (NADPH) as a reducing agent [79]. Resulting GS-SeH is converted to SeCys by its coupling with O-acetylserine (OAS). SeCys can additionally be formed directly from Se(IV) by the action of selenomethyltransferase (SMT) [92].

### 6.3. Metabolic Fates of Selenocysteine

SeCys is the starting substrate for the synthesis of other selenocompounds, including SeMet. In algae, as in plants, SeCys can also be converted to elemental Se (Se^0^) and alanine by the activity of SeCyslyase (SL). Because Se^0^ does not significantly interfere with most cellular metabolic reactions, its production provides these organisms with increased tolerance to Se [14,91]. Another possible fate of SeCys is its methylation catalyzed by the enzyme SeCys methyltransferase (SeCysMT) to produce methylselenocysteine (MeSeCys) [14,80,93]. *C. reinhardtii*, in particular, has been reported to methylate up to 89% of intracellular Se [49], and MeSeCys has been detected in the unicellular oceanic microalga *Emiliania huxleyi* [94]. Specifically, Se metabolites in *E. huxleyi* were dissected using thin-layer chromatography (TLC) and radio-luminography. Results evidenced about 70% incorporation of ^75^Se in the low molecular mass fraction of cellular components (LMCs) compared to the protein fraction (about 17%) after 16 h incubation of the microalga with labeled Se(IV). Among LMCs, only MeSeCys was retrieved, while Se(IV), SeCys, and SeMet were not detected. A conceivable hypothesis is that Se amino acids in *E. huxleyi* are promptly assimilated into non-toxic intermediates as a mechanism of Se detoxification [94]. However, because ^75^Se was distinctly recorded in the protein fraction, it is likely that Se amino acids were not directly inserted into proteins during their synthesis, but initially converted to ^75^Se^2−^ and then incorporated into selenoproteins through their de novo synthesis.

### 6.4. Metabolic Fates of Methylselenocysteine

MeSeCys in plants is known to have various possible metabolic fates, like its conversion into S-methylselenoglutathione (S-MeSeGS) by the enzyme methionine-γ-lyase, or its conjugation with glutamate to form γ-glutamylmethyl-SeCys in a reaction mediated by glutamylcysteinesynthetase [95,96,97]. MeSeCys can also be converted into volatile dimethyldiselenide (DMDSe), and this compound was detected in both plants and algae [14,49,98]. In addition to DMDSe, algae can produce the volatile compound dimethylselenide (DMSe) in the cytosol through a sequential process that starts from SeCys and produces seleniumhomocysteine (SeHCys), SeMet, and methyl-SeMet (MeSeMet) as intermediates in reactions catalyzed by the enzymes cystathionine-β-lyase, methioninesynthase, and methioninemethyltransferase (MMT), respectively [80,91]. MeSeMet is converted into DMSe by two possible processes: it can be converted into dimethylselenopropionate (DMSeP) by betainealdehydrogenase and finally into DMSe by di-methylselenopropionate lyase, or it is directly transformed to DMSe by the activity of methylmethionine hydrolase [99]. *C. reinhardtii* and *Chlorella* sp. ably produce both DMDSe and DMSe, with DMDSe prevalent over DMSe [49,100]. Overall, Se volatilization appears to be a more efficient process in microalgae than in plants, and thus it is deemed as a key process in the Se-cycling through the ecosystem [49,100]. Appreciable Se volatilization has also been reported for macroalgae [101]. 

### 6.5. SeCys Insertion into Selenoproteins

The metabolic routes involving the synthesis of Se^0^, volatile DMSe and DMDSe, and Se organic compounds from SeCys and SeMet, have the main function to reduce Se toxicity by preventing Se amino acids’ misincorporation into proteins [14,102,103]. Indeed, although several microalgae possess a specific mechanism for the synthesis of essential selenoproteins, Se can determine its toxicity in these organisms when accumulated in excess due to the unwanted replacement of Met with SeMet in proteins. The mechanism used by microalgae for selenoprotein synthesis is complex, but very similar to that discovered in humans and mammals [83,104]. It consists in the translational insertion of SeCys at the catalytic site of selenoproteins using a specific t-RNA (SeCys-tRNA([Ser]Sec), whose anticodon recognizes an opal (UGA) stop codon as a SeCys codon [105,106]. The recoding of the UGA stop codon is dictated by a SeCys insertion sequence (SECIS) localized at the 3′ untranslated region (UTR) of selenoprotein genes [87,107,108]. Genes containing SECIS sequences have high sequence similarity among algae and animals, and likely share a common origin. In support of this, genes coding for selenoproteins in *C. reinhardtii* can efficiently direct the synthesis of selenoproteins once expressed in mammalian cells [83]. SeCys-tRNA inserts SeCys into selenoproteins with the help of two other players, a Sec-specific elongation factor (EFsec) and a SECIS-binding protein 2 [108].

### 6.6. Selenoproteome

Large selenoproteomes seem to be associated with the aquatic life, as microalgae and other aquatic organisms are generally rich in selenoproteins [87]. For algae, selenoproteins were first identified in *C. reinhardtii* [83,109], and later in other unicellular algae, including *Ostreococcus* (Prasinophyceae) [84,110], *Cyanidioschyzon* (Cyanidiaceae) [111], and *Emiliania huxleyi* (Haptophytes) [112], mainly by means of advanced bioinformatics tools and radio-labeling techniques using ^75^Se. *Ostreococcus tauri* and *Ostreococcus lucimarinus*, in particular, possess more selenoproteins than mammalian cells, encoded by 26 and 29 selenoprotein genes, respectively [84]. In *O. tauri*, 14 selenoproteins show high sequence homology to human selenoproteins, such as thioredoxin reductase (TR), SelT, SelM, SelK, SelS, Sep15, SelO, SelH, SelW1-2, and five glutathione peroxidase (GPx) [84]. Five genes in this microalga were also homologs of other eukaryotic selenoproteins (MsrA, SelU, and three disulfide isomerase (PDI) homologs) and three were homologs of bacterial selenoproteins (methyltransferase, thioredoxin-fold protein, and peroxiredoxin) [84]. Further selenoprotein genes have been discovered in *O. tauri*, but their function is still unknown [84]. In *C. reinhardtii*, at least ten selenoproteins have been identified, including two homologs of mammalian phospholipid hydroperoxide glutathione peroxidase (PHGPx1 and PHGPx2), two homologs of selenoprotein W (SelW1 and SelW2), a methionine-S-sulfoxide reductase (MsrA1), two homologs of mammalian selenoprotein M (SelM1 and SelM2), a homolog of mammalian selenoprotein T (SelT1), a selenoprotein K homolog (SelK1), and a homolog of mammalian thioredoxin reductase (TR1) [83,113,114]. In the marine-centric diatom *Thalassiosira pseudonana*, 16 selenoprotein genes have been detected, which encode two GPx homologs, SelT, TR, SPS2, two SelM, two SelU, MsrA, two PDI homologs, a predicted SAM-dependent methyltransferase, two peroxiredoxins, and one thioredoxin-like protein [84]. Six selenoproteins have also been reported for the marine coccolithophorid *E. huxleyi* [94,115]. This microalga holds two major selenoproteins, i.e., a thioredoxin reductase [112] and a protein-disulfide isomerase (PDI)-like protein, whose role consists in controlling the conformational changes and folding of proteins that mainly function in the endoplasmic reticulum by acting on their disulfide bonds [115]. In the unicellular red alga *Cyanidioschyzon merolae*, the various components of the SeCys insertion machinery have been revealed, but no candidate selenoprotein has been identified [84]. In macroalgae, no selenoproteosome has been described so far. This means that although Se concentrations in their environment are steady, macroalgae likely lost the Se essential metabolism during evolution for reasons that are not clear yet.

## 7. Final Remarks

### 7.1. Selenium Risk Assessment

Algae are very resistant to Se, possibly due in part to the use of Se in metabolic processes and the ability to sequester or excrete Se if necessary [26]. Selenium toxicity to algae was not a focus of this review, since toxic concentrations are much higher than those encountered in natural or impacted aquatic ecosystems. On the other hand, it was very important to further our understanding of algae bioaccumulation across aquatic systems because of the importance of dietary transfer of Se from algae to higher trophic levels. Algae Se bioaccumulation was highly variable as a function of water [TSe], but grouping lotic and lentic systems provided a means to reduce this variability. Grouping particulate types did not appear to help in understanding this variability. Using our model (Figure 1B) in ecological risk assessments could help to better estimate Se bioaccumulation for lentic and lotic systems. Generally, water [Se(VI)] were higher than those of other Se species in lotic systems. A competition model for the interaction of Se(VI) and SO_4_^2−^ has been created and could be used in the future for lotic Se risk assessment, if SO_4_^2−^ is measured (and ideally Se(VI)). Our review showed that Se(IV) bioaccumulation does not reach a plateau at high concentrations and is less affected than Se(VI) by [SO_4_^2−^], which helps explain why the uptake of Se(IV) is generally perceived as higher than that of Se(VI). When evaluating ecotoxicological risks of Se released into lotic systems, we propose to perform the risk assessment in the nearest downstream lentic receiving waters, because these lentic waterbodies will have longer residence times and biogeochemical processes that favor Se assimilation into food webs.

### 7.2. Recommendations for Future Studies

There are still many gaps in the knowledge of Se uptake by aquatic microorganisms. In order to address and minimize some of the variability in Se uptake, and to better understand the patterns of Se uptake in algae and other organisms at the base of the food web, several chemical and physical characteristics of study systems and study organisms should be reported. Water chemistry characteristics, including the pH and [SO_4_^2−^] concentrations in a waterbody, should be measured in field and lab studies aimed at characterizing Se uptake in organisms. Also, speciation of Se and the characteristics of a study system, including residence time and watershed size, will be important in determining the species of Se present. There should be some standardization of samples used to represent the base of the food web, and some taxonomic characterization of samples. For instance, if samples are collected from sediment, sediment depth, organic matter content, and particle size are essential reporting requirements. For water column particulates or bulk phytoplankton, total suspended solids, particle size classes, and chlorophyll *a* content should be measured. For periphyton or biofilm samples, chlorophyll *a* and ash-free dry mass can give a measure of relative amounts of algae and organic matter. Further characterization of the taxa present, including algae, bacteria, fungi, and protozoa could contribute significantly to our understanding of Se uptake.

Regarding Se species uptake, there are very few studies on the characterization of Se species in natural waters and their uptake by aquatic microorganisms. We should better characterize org-Se uptake at several concentrations for several taxa including bacteria, fungi, and algae. Studies on Se(IV) uptake are clearly important as well and, to date, only one study reported an effect of pH on algal uptake. The Se(IV) competition model gave mitigated results, in part because of the lack of Se(IV) and SO_4_^2−^ interaction studies.

## Figures and Tables

**Table 1:** Overall effect of an increase (↑) in pH, sulfate (SO_4_^2−^), or phosphate (PO_4_^3−^) on the uptake of selenate (Se(VI), selenite (Se(IV)), or selenomethionine (SeMet); ↑ = increase in uptake; ↓ = decrease in uptake; - = no effect; ? = contradictory or no observations.

Se Species	↑ pH	↑ SO_4_^2−^	↑ PO_4_^3−^
Se(VI)	-	↓↓↓	?
Se(IV)	↑↑	↓	↓
SeMet	↑	-	?

## Supplementary Materials

Table S1, S2, and S3 (see details in Section 2) are presented in this Supplementary Material. The following are available online at https://www.mdpi.com/2223-7747/9/4/528/s1, Table S1: Water and particulate selenium concentrations in field and laboratory studies, Table S2: Water total dissolved selenium concentrations and speciation. Table S3: Algae Se concentrations at different aqueous selenite, selenate, and sulfate exposure concentrations.
