# Supplementary material for: Selenium Interactions with Algae: Chemical Processes at Biological Uptake Sites, Bioaccumulation, and Intracellular Metabolism
Source: Plants (Basel). 2020 Apr 19;9(4):528. doi: 10.3390/plants9040528 (PMC7238072; doi:10.3390/plants9040528)
Supplement: Supplementary file 1 [file plants-09-00528-s001.pdf]

Supplementary Information

Selenium Interactions with Algae: Chemical Processes at Biological Uptake Sites, Bioaccumulation, and Intracellular Metabolism

Dominic E. Ponton [1,*], Stephanie D. Graves [2], Claude Fortin [3], David Janz [4], Marc Amyot [1], and Michela Schiavon [5]

[1] GRIL, Université de Montréal, Département des Sciences Biologiques, Montreal, Quebec, Canada, H2V 0B3
[2] University of Saskatchewan, Toxicology Graduate Program, Saskatoon, Saskatchewan, Canada, S7N 5B3
[3] EcotoQ, Institut National de la Recherche Scientifique, Centre Eau, Terre et Environnement, Quebec, Canada, G1K 9A9
[4] University of Saskatchewan, Department of Veterinary Biomedical Sciences, Saskatoon, Saskatchewan, Canada, S7N 5B4
[5] Università degli Studi di Padova, DAFNAE Department, Legnaro, Italy, 35020
* Correspondence: dominic.ponton@umontreal.ca

Keywords: algae; selenium; competition; sulfate; partition coefficient; selenate; selenite; organic selenium

This document contains three tables:

Table S1: Water and particulate selenium concentrations in field and laboratory studies.

Table S2: Water total dissolved selenium concentrations and speciation.

Table S3: Algae Se concentrations at different aqueous selenite, selenate, and sulphate exposure concentrations.

Table S1: Water and particulate selenium concentrations in field and laboratory studies

Site	Sample Type	System	SO4 (mg/L)	Total dissolved Se water (μg/L)	Particulate Se (ug/g dw)	kd (L/kg)	Reference
San Francisco Bay, California (1998-1999)	NA	Estuary	NA	0.1	0.8	7219	Cutter and Cutter 2004; Doblin et al., 2006
Salton Sea estuary, Alamo River	Suspended particulates	Estuary	NA	0.9	8.7	9457	LeBlanc and Schroeder, 2008
Salton Sea estuary, Whitewater River	Suspended particulates	Estuary	NA	0.9	11.0	11957	LeBlanc and Schroeder, 2008
Outer San Francisco Bay, California (1998-1999)	NA	Estuary	NA	0.1	1.2	13636	Cutter and Cutter 2004; Doblin et al., 2006
Xiamen Bay, Fujian Province, China	NA	Estuary	NA	0.1	1.2	15000	Liu et al., 1987
Salton Sea estuary, New River, California	Suspended particulates	Estuary	NA	0.9	16.0	17391	LeBlanc and Schroeder, 2008
San Francisco Bay (1986;1995-96) (range 3,000-40,000)	NA	Estuary	NA	0.2	4.2	18444	Presser and Luoma, 2006
Lower Newport Bay, California (range 6,933-42,715)	Suspended particulates	Estuary	NA	0.1	2.0	18318	Presser and Luoma, 2009
Laboratory	*Anabaena flos-aquae* (cyanobacterium)	LabSeIV	2.7	1030.0	271.0	263	Kiffney and Knight 1990
Laboratory	*Anabaena flos-aquae* (cyanobacterium)	LabSeIV	2.7	2920.0	1071.0	367	Kiffney and Knight 1990
Laboratory	*Anabaena flos-aquae* (cyanobacterium)	LabSeIV	2.7	4990.0	6072.0	1217	Kiffney and Knight 1990
Laboratory	*Anabaena flos-aquae* (cyanobacterium)	LabSeIV	2.7	9870.0	9365.0	949	Kiffney and Knight 1990
Laboratory	*Chlamydomonas reinhardtii* (green alga)	LabSeIV	60.0	0.8	2.1	2515	Besser et al. 1993
Laboratory	*Chlamydomonas reinhardtii* (green alga)	LabSeIV	60.0	8.6	18.6	2175	Besser et al. 1993
Laboratory	*Chlamydomonas reinhardtii* (green alga)	LabSeIV	60.0	95.2	46.1	484	Besser et al. 1993
Laboratory	*Scenedesmus obliquus* (green alga)	LabSeIV	NA	5.4	1.1	194	Guan and Wang 2004
Laboratory	*Scenedesmus obliquus* (green alga)	LabSeIV	NA	52.2	5.3	101	Guan and Wang 2004
Laboratory	*Scenedesmus obliquus* (green alga)	LabSeIV	NA	374.0	21.1	56	Guan and Wang 2004
Laboratory	*Scenedesmus obliquus* (green alga)	LabSeIV	NA	500.0	105.3	211	Guan and Wang 2004
Laboratory	*Chlamydomonas reinhardtii* (green alga)	LabSeIV	NA	1.4	1.1	750	Guan and Wang 2004
Laboratory	*Chlamydomonas reinhardtii* (green alga)	LabSeIV	NA	4.2	5.3	1254	Guan and Wang 2004
Laboratory	*Chlamydomonas reinhardtii* (green alga)	LabSeIV	NA	9.7	21.1	2173	Guan and Wang 2004
Laboratory	*Chlamydomonas reinhardtii* (green alga)	LabSeIV	NA	30.6	105.3	3440	Guan and Wang 2004
Laboratory	Natural periphyton biofilms	LabSeIV	41.0	2.4	2.0	846	Conley et al. 2009
Laboratory	Natural periphyton biofilms	LabSeIV	41.0	2.4	2.1	863	Conley et al. 2009
Laboratory	Natural periphyton biofilms	LabSeIV	41.0	4.9	4.3	880	Conley et al. 2009
Laboratory	Natural periphyton biofilms	LabSeIV	41.0	10.7	12.3	1146	Conley et al. 2009
Laboratory	Natural periphyton biofilms	LabSeIV	41.0	10.3	9.4	912	Conley et al. 2009
Laboratory	Natural periphyton biofilms	LabSeIV	41.0	12.6	24.8	1968	Conley et al. 2009
Laboratory	Natural periphyton biofilms	LabSeIV	41.0	13.9	21.5	1547	Conley et al. 2009
Laboratory	Natural periphyton biofilms	LabSeIV	41.0	1.1	4.3	3909	Conley et al. 2011
Laboratory	Natural periphyton biofilms	LabSeIV	41.0	5.9	15.1	2559	Conley et al. 2011
Laboratory	Natural periphyton biofilms	LabSeIV	41.0	21.4	30.9	1444	Conley et al. 2011
Laboratory	Natural periphyton biofilms	LabSeIV	41.0	2.7	11.2	4148	Conley et al. 2011
Laboratory	Natural periphyton biofilms	LabSeIV	41.0	7.1	21.2	2986	Conley et al. 2011
Laboratory	Natural periphyton biofilms	LabSeIV	41.0	19.2	45.3	2359	Conley et al. 2011
Laboratory	Natural periphyton biofilms	LabSeIV	41.0	3.4	10.3	3029	Conley et al. 2011
Laboratory	Natural periphyton biofilms	LabSeIV	41.0	8.9	23.0	2584	Conley et al. 2011
Laboratory	Natural periphyton biofilms	LabSeIV	41.0	23.1	50.7	2195	Conley et al. 2011
Laboratory	*Lemna minor* (duckweed)	LabSeIV	NA	0.5	0.2	392	Nautilus 2013a
Laboratory	*Lemna minor* (duckweed)	LabSeIV	NA	10.5	3.0	283	Nautilus 2013a
Laboratory	*Lemna minor* (duckweed)	LabSeIV	NA	20.5	4.8	234	Nautilus 2013a
Laboratory	*Lemna minor* (duckweed)	LabSeIV	NA	41.3	9.8	237	Nautilus 2013a
Laboratory	*Lemna minor* (duckweed)	LabSeIV	187.0	12.9	4.1	315	Nautilus 2013a
Laboratory	*Lemna minor* (duckweed)	LabSeIV	NA	11.6	3.9	335	Nautilus 2013a
Laboratory	*Lemna minor* (duckweed)	LabSeIV	NA	10.3	1.9	188	Nautilus 2013a
Laboratory	*Lemna minor* (duckweed)	LabSeIV	NA	10.3	3.0	290	Nautilus 2013a
Laboratory	Scenedesmus quadricauda	LabSeIV	384.2	10000.0	689.0	69	Umysova et al. 2009
Laboratory	Scenedesmus quadricauda	LabSeIV	3842.4	10000.0	678.0	68	Umysova et al. 2009
Laboratory	Scenedesmus quadricauda	LabSeIV	38424.0	10000.0	706.0	71	Umysova et al. 2009
Laboratory	Scenedesmus quadricauda	LabSeIV	3842.4	50000.0	3730.0	75	Umysova et al. 2009
Laboratory	Scenedesmus quadricauda	LabSeIV	38424.0	50000.0	3500.0	70	Umysova et al. 2009
Laboratory	Scenedesmus quadricauda	LabSeIV	384.2	50000.0	1433.0	29	Vitova et al. 2011
Laboratory	Chlamydomonas reinhardtii	LabSeIV	0.0	3.9	41.3	10469	Morlon et al. 2006
Laboratory	Chlamydomonas reinhardtii	LabSeIV	0.0	3.9	31.8	8053	Morlon et al. 2006
Laboratory	Chlamydomonas reinhardtii	LabSeIV	0.1	3.9	17.9	4535	Morlon et al. 2006
Laboratory	Chlamydomonas reinhardtii	LabSeIV	0.8	3.9	6.5	1653	Morlon et al. 2006
Laboratory	Chlamydomonas reinhardtii	LabSeIV	7.7	3.9	5.9	1483	Morlon et al. 2006
Laboratory	Chlamydomonas reinhardtii	LabSeIV	76.8	3.9	2.0	509	Morlon et al. 2006
Laboratory	Chlamydomonas reinhardtii	LabSeIV	0.0	3.9	48.0	12165	Morlon et al. 2006
Laboratory	Chlamydomonas reinhardtii	LabSeIV	0.0	3.9	41.3	10469	Morlon et al. 2006
Laboratory	Chlamydomonas reinhardtii	LabSeIV	0.1	3.9	25.3	6400	Morlon et al. 2006
Laboratory	Chlamydomonas reinhardtii	LabSeIV	0.8	3.9	22.1	5595	Morlon et al. 2006
Laboratory	Chlamydomonas reinhardtii	LabSeIV	7.7	3.9	15.4	3899	Morlon et al. 2006
Laboratory	Chlamydomonas reinhardtii	LabSeIV	76.8	3.9	7.4	1865	Morlon et al. 2006
Laboratory	Chlamydomonas reinhardtii	LabSeIV	0.0	5.0	3.9	778	Ponton et al. 2018
Laboratory	Chlamydomonas reinhardtii	LabSeIV	0.0	5.0	3.3	665	Ponton et al. 2018
Laboratory	Chlamydomonas reinhardtii	LabSeIV	0.1	5.0	1.6	314	Ponton et al. 2018
Laboratory	Chlamydomonas reinhardtii	LabSeIV	0.3	5.0	1.3	258	Ponton et al. 2018
Laboratory	Chlamydomonas reinhardtii	LabSeIV	0.6	5.0	1.4	283	Ponton et al. 2018
Laboratory	Chlamydomonas reinhardtii	LabSeIV	5.8	5.0	1.3	253	Ponton et al. 2018
Laboratory	Chlamydomonas reinhardtii	LabSeIV	9.6	5.0	1.3	256	Ponton et al. 2018
Laboratory	Chlamydomonas reinhardtii	LabSeIV	19.2	5.0	1.2	248	Ponton et al. 2018
Laboratory	Chlamydomonas reinhardtii	LabSeIV	76.8	5.0	1.3	264	Ponton et al. 2018
Laboratory	Chlamydomonas reinhardtii	LabSeIV	153.7	5.0	1.1	225	Ponton et al. 2018
Laboratory	Chrysophyceae, Dynophyceae, Euglenophyceae, bacteria	LabSeIV	0.0	5.0	56.5	11356	Ponton et al. 2018
Laboratory	Chrysophyceae, Dynophyceae, Euglenophyceae, bacteria	LabSeIV	0.3	5.0	55.8	11215	Ponton et al. 2018
Laboratory	Chrysophyceae, Dynophyceae, Euglenophyceae, bacteria	LabSeIV	2.9	5.0	51.2	10299	Ponton et al. 2018
Laboratory	Chrysophyceae, Dynophyceae, Euglenophyceae, bacteria	LabSeIV	24.1	5.0	44.6	8963	Ponton et al. 2018
Laboratory	Chrysophyceae, Dynophyceae, Euglenophyceae, bacteria	LabSeIV	288.2	5.0	47.0	9440	Ponton et al. 2018
Laboratory	Arthrospira platensis	LabSeIV	634.0	10.0	22.0	2200	Li et al. 2003
Laboratory	Arthrospira platensis	LabSeIV	634.0	400.0	219.0	548	Li et al. 2003
Laboratory	Chlorophyta (natural periphyton)	LabSeIV	2.9	5.0	33.1	6620	Markwart et al. 2018
Laboratory	Bacillariophyta (natural periphyton)	LabSeIV	3.8	5.0	7.3	1460	Markwart et al. 2018
Laboratory	Chlorophyta/Bacillariophyta (natural periphyton)	LabSeIV	5.8	5.0	22.6	4520	Markwart et al. 2018
Laboratory	Chlorophyta	LabSeIV	2.9	5.0	33.7	6740	Markwart et al. 2018
Laboratory	Cyanophyta	LabSeIV	0.3	5.0	119.1	23820	Markwart et al. 2018
Laboratory	Chlorophyta (natural periphyton)	LabSeIV	2.9	25.0	46.9	1876	Markwart et al. 2018
Laboratory	Bacillariophyta (natural periphyton)	LabSeIV	3.8	25.0	16.8	672	Markwart et al. 2018
Laboratory	Chlorophyta/Bacillariophyta (natural periphyton)	LabSeIV	5.8	25.0	49.7	1988	Markwart et al. 2018
Laboratory	Chlorophyta	LabSeIV	2.9	25.0	65.7	2628	Markwart et al. 2018
Laboratory	Cyanophyta	LabSeIV	0.3	25.0	222.5	8900	Markwart et al. 2018
Laboratory	Chlamydomonas reinhardtii	LabSeIV	4.6	10.0	19.7	1965	Riedel and Sanders 1996
Laboratory	Chlamydomonas reinhardtii	LabSeIV	9.4	10.0	12.1	1211	Riedel and Sanders 1996
Laboratory	Chlamydomonas reinhardtii	LabSeIV	19.0	10.0	10.9	1090	Riedel and Sanders 1996
Laboratory	Chlamydomonas reinhardtii	LabSeIV	47.7	10.0	9.9	987	Riedel and Sanders 1996
Laboratory	Chlamydomonas reinhardtii	LabSeIV	96.2	10.0	7.1	710	Riedel and Sanders 1996
Laboratory	Chlamydomonas reinhardtii	LabSeIV	4.6	3.0	9.4	3117	Riedel and Sanders 1996
Laboratory	Chlamydomonas reinhardtii	LabSeIV	9.4	3.0	6.5	2159	Riedel and Sanders 1996
Laboratory	Chlamydomonas reinhardtii	LabSeIV	19.0	3.0	6.9	2304	Riedel and Sanders 1996
Laboratory	Chlamydomonas reinhardtii	LabSeIV	47.7	3.0	5.5	1824	Riedel and Sanders 1996
Laboratory	Chlamydomonas reinhardtii	LabSeIV	96.0	3.0	4.2	1404	Riedel and Sanders 1996
Laboratory	*Anabaena flos-aquae* (cyanobacterium)	LabSeVI	2.7	995.0	30.4	31	Kiffney and Knight 1990
Laboratory	*Anabaena flos-aquae* (cyanobacterium)	LabSeVI	2.7	2990.0	744.0	249	Kiffney and Knight 1990
Laboratory	*Anabaena flos-aquae* (cyanobacterium)	LabSeVI	2.7	4930.0	1218.0	247	Kiffney and Knight 1990
Laboratory	*Anabaena flos-aquae* (cyanobacterium)	LabSeVI	2.7	9720.0	1313.0	135	Kiffney and Knight 1990
Laboratory	*Chlamydomonas reinhardtii* (green alga)	LabSeVI	60.0	9.5	3.7	389	Besser et al. 1993
Laboratory	*Chlamydomonas reinhardtii* (green alga)	LabSeVI	60.0	92.1	31.1	338	Besser et al. 1993
Laboratory	*Chlamydomonas reinhardtii* (green alga)	LabSeVI	60.0	934.0	367.0	393	Besser et al. 1993
Laboratory	*Chlorella pyrenoidosa* (green alga)	LabSeVI	3.8	2500.0	3100.0	1240	Bennett et al. 1986
Laboratory	*Chlorella pyrenoidosa* (green alga)	LabSeVI	3.8	2500.0	2600.0	1040	Bennett et al. 1986
Laboratory	*Selenastrum capricornutum* (green alga)	LabSeVI	14.4	4.0	1.1	275	Malchow et al. 1995
Laboratory	*Selenastrum capricornutum* (green alga)	LabSeVI	14.4	10.0	2.1	211	Malchow et al. 1995

Laboratory	*Selenastrum capricornutum* (green alga)	LabSeVI	14.4	40.0	7.2	179	Malchow et al. 1995
Laboratory	*Selenastrum capricornutum* (green alga)	LabSeVI	3.3	11.3	17.0	1504	Williams et al. 1994
Laboratory	*Selenastrum capricornutum* (green alga)	LabSeVI	3.3	107.0	156.0	1458	Williams et al. 1994
Laboratory	*Selenastrum capricornutum* (green alga)	LabSeVI	33.0	11.3	4.0	354	Williams et al. 1994
Laboratory	*Selenastrum capricornutum* (green alga)	LabSeVI	33.0	107.0	11.0	103	Williams et al. 1994
Laboratory	*Chlorella vulgaris* (green alga)	LabSeVI	14.4	105.5	35.0	332	Dobbs et al. 1996
Laboratory	*Chlorella vulgaris* (green alga)	LabSeVI	14.4	204.9	75.0	366	Dobbs et al. 1996
Laboratory	*Chlorella vulgaris* (green alga)	LabSeVI	14.4	397.6	155.0	390	Dobbs et al. 1996
Laboratory	Blue-green algae mixture	LabSeVI	NA	7.3	6.3	861	Thomas et al. 1999
Laboratory	Blue-green algae mixture	LabSeVI	NA	87.0	36.6	420	Thomas et al. 1999
Laboratory	Blue-green algae mixture	LabSeVI	NA	870.0	111.0	128	Thomas et al. 1999
Laboratory	*Pseudokirchneriella subcapitata* (green alga)	LabSeVI	17.0	0.5	1.2	2521	Rickwood and Jatar 2013
Laboratory	*Pseudokirchneriella subcapitata* (green alga)	LabSeVI	17.0	1.1	0.7	661	Rickwood and Jatar 2013
Laboratory	*Pseudokirchneriella subcapitata* (green alga)	LabSeVI	17.0	1.3	2.2	1719	Rickwood and Jatar 2013
Laboratory	*Pseudokirchneriella subcapitata* (green alga)	LabSeVI	17.0	1.9	1.2	660	Rickwood and Jatar 2013
Laboratory	*Pseudokirchneriella subcapitata* (green alga)	LabSeVI	17.0	2.6	2.9	1114	Rickwood and Jatar 2013
Laboratory	*Pseudokirchneriella subcapitata* (green alga)	LabSeVI	17.0	2.7	2.1	793	Rickwood and Jatar 2013
Laboratory	*Pseudokirchneriella subcapitata* (green alga)	LabSeVI	17.0	3.4	4.3	1256	Rickwood and Jatar 2013
Laboratory	*Pseudokirchneriella subcapitata* (green alga)	LabSeVI	17.0	5.0	2.8	563	Rickwood and Jatar 2013
Laboratory	*Pseudokirchneriella subcapitata* (green alga)	LabSeVI	17.0	6.5	4.0	621	Rickwood and Jatar 2013
Laboratory	*Pseudokirchneriella subcapitata* (green alga)	LabSeVI	17.0	7.7	8.0	1036	Rickwood and Jatar 2013
Laboratory	*Pseudokirchneriella subcapitata* (green alga)	LabSeVI	17.0	8.0	2.3	293	Rickwood and Jatar 2013
Laboratory	*Pseudokirchneriella subcapitata* (green alga)	LabSeVI	17.0	10.3	16.6	1603	Rickwood and Jatar 2013
Laboratory	*Pseudokirchneriella subcapitata* (green alga)	LabSeVI	17.0	15.1	14.6	969	Rickwood and Jatar 2013
Laboratory	*Pseudokirchneriella subcapitata* (green alga)	LabSeVI	17.0	15.8	7.8	491	Rickwood and Jatar 2013
Laboratory	*Pseudokirchneriella subcapitata* (green alga)	LabSeVI	17.0	21.0	32.4	1542	Rickwood and Jatar 2013
Laboratory	*Pseudokirchneriella subcapitata* (green alga)	LabSeVI	17.0	27.2	16.2	596	Rickwood and Jatar 2013
Laboratory	*Pseudokirchneriella subcapitata* (green alga)	LabSeVI	17.0	30.4	41.6	1367	Rickwood and Jatar 2013
Laboratory	*Pseudokirchneriella subcapitata* (green alga)	LabSeVI	17.0	40.9	29.3	717	Rickwood and Jatar 2013
Laboratory	*Pseudokirchneriella subcapitata* (green alga)	LabSeVI	17.0	41.1	54.2	1319	Rickwood and Jatar 2013
Laboratory	*Pseudokirchneriella subcapitata* (green alga)	LabSeVI	17.0	41.4	33.3	805	Rickwood and Jatar 2013
Laboratory	*Pseudokirchneriella subcapitata* (green alga)	LabSeVI	17.0	43.2	24.9	576	Rickwood and Jatar 2013
Laboratory	*Pseudokirchneriella subcapitata* (green alga)	LabSeVI	17.0	43.5	37.5	862	Rickwood and Jatar 2013
Laboratory	*Pseudokirchneriella subcapitata* (green alga)	LabSeVI	17.0	50.5	20.2	400	Rickwood and Jatar 2013
Laboratory	*Pseudokirchneriella subcapitata* (green alga)	LabSeVI	17.0	52.1	46.6	894	Rickwood and Jatar 2013
Laboratory	*Pseudokirchneriella subcapitata* (green alga)	LabSeVI	17.0	52.3	25.3	483	Rickwood and Jatar 2013
Laboratory	*Pseudokirchneriella subcapitata* (green alga)	LabSeVI	17.0	52.3	28.1	537	Rickwood and Jatar 2013
Laboratory	*Pseudokirchneriella subcapitata* (green alga)	LabSeVI	17.0	60.2	184.4	3063	Rickwood and Jatar 2013
Laboratory	*Pseudokirchneriella subcapitata* (green alga)	LabSeVI	17.0	101.0	98.5	976	Rickwood and Jatar 2013
Laboratory	*Pseudokirchneriella subcapitata* (green alga)	LabSeVI	17.0	106.5	91.6	860	Rickwood and Jatar 2013
Laboratory	*Pseudokirchneriella subcapitata* (green alga)	LabSeVI	17.0	107.1	96.3	899	Rickwood and Jatar 2013
Laboratory	*Pseudokirchneriella subcapitata* (green alga)	LabSeVI	17.0	123.0	314.3	2555	Rickwood and Jatar 2013
Laboratory	*Chlorella vulgaris* (green alga)	LabSeVI	17.0	0.5	1.4	2672	Rickwood and Jatar 2013
Laboratory	*Chlorella vulgaris* (green alga)	LabSeVI	17.0	1.1	2.3	2052	Rickwood and Jatar 2013
Laboratory	*Chlorella vulgaris* (green alga)	LabSeVI	17.0	1.6	0.2	120	Rickwood and Jatar 2013
Laboratory	*Chlorella vulgaris* (green alga)	LabSeVI	17.0	2.8	7.5	2720	Rickwood and Jatar 2013
Laboratory	*Chlorella vulgaris* (green alga)	LabSeVI	17.0	4.0	0.5	122	Rickwood and Jatar 2013
Laboratory	*Chlorella vulgaris* (green alga)	LabSeVI	17.0	6.1	5.5	902	Rickwood and Jatar 2013
Laboratory	*Chlorella vulgaris* (green alga)	LabSeVI	17.0	6.7	4.4	656	Rickwood and Jatar 2013
Laboratory	*Chlorella vulgaris* (green alga)	LabSeVI	17.0	7.2	1.5	204	Rickwood and Jatar 2013
Laboratory	*Chlorella vulgaris* (green alga)	LabSeVI	17.0	9.4	1.2	126	Rickwood and Jatar 2013
Laboratory	*Chlorella vulgaris* (green alga)	LabSeVI	17.0	10.1	7.4	727	Rickwood and Jatar 2013
Laboratory	*Chlorella vulgaris* (green alga)	LabSeVI	17.0	12.1	2.6	216	Rickwood and Jatar 2013
Laboratory	*Chlorella vulgaris* (green alga)	LabSeVI	17.0	16.2	6.9	426	Rickwood and Jatar 2013
Laboratory	*Chlorella vulgaris* (green alga)	LabSeVI	17.0	19.5	14.4	739	Rickwood and Jatar 2013
Laboratory	*Chlorella vulgaris* (green alga)	LabSeVI	17.0	22.1	24.5	1109	Rickwood and Jatar 2013
Laboratory	*Chlorella vulgaris* (green alga)	LabSeVI	17.0	24.5	5.0	204	Rickwood and Jatar 2013
Laboratory	*Chlorella vulgaris* (green alga)	LabSeVI	17.0	38.6	39.1	1014	Rickwood and Jatar 2013
Laboratory	*Chlorella vulgaris* (green alga)	LabSeVI	17.0	39.2	27.0	689	Rickwood and Jatar 2013
Laboratory	*Chlorella vulgaris* (green alga)	LabSeVI	17.0	41.4	14.0	339	Rickwood and Jatar 2013
Laboratory	*Chlorella vulgaris* (green alga)	LabSeVI	17.0	42.1	35.7	848	Rickwood and Jatar 2013
Laboratory	*Chlorella vulgaris* (green alga)	LabSeVI	17.0	42.4	13.0	306	Rickwood and Jatar 2013
Laboratory	*Chlorella vulgaris* (green alga)	LabSeVI	17.0	46.8	7.9	168	Rickwood and Jatar 2013
Laboratory	*Chlorella vulgaris* (green alga)	LabSeVI	17.0	46.8	9.5	203	Rickwood and Jatar 2013
Laboratory	*Chlorella vulgaris* (green alga)	LabSeVI	17.0	46.9	10.6	227	Rickwood and Jatar 2013
Laboratory	*Chlorella vulgaris* (green alga)	LabSeVI	17.0	47.2	22.1	469	Rickwood and Jatar 2013
Laboratory	*Chlorella vulgaris* (green alga)	LabSeVI	17.0	91.4	159.4	1744	Rickwood and Jatar 2013
Laboratory	*Chlorella vulgaris* (green alga)	LabSeVI	17.0	93.1	44.7	481	Rickwood and Jatar 2013
Laboratory	*Chlorella vulgaris* (green alga)	LabSeVI	17.0	103.5	68.3	660	Rickwood and Jatar 2013
Laboratory	*Chlorella vulgaris* (green alga)	LabSeVI	17.0	103.6	52.9	511	Rickwood and Jatar 2013
Laboratory	*Chlorella vulgaris* (green alga)	LabSeVI	17.0	108.2	78.5	726	Rickwood and Jatar 2013
Laboratory	Chlamydomonas reinhardtii	LabSeVI	0.8	0.8	16.4	20770	Fournier et al. 2010
Laboratory	Chlamydomonas reinhardtii	LabSeVI	0.8	7.9	56.5	7156	Fournier et al. 2010
Laboratory	Chlamydomonas reinhardtii	LabSeVI	0.8	47.4	889.2	18769	Fournier et al. 2010
Laboratory	Chlamydomonas reinhardtii	LabSeVI	0.8	197.4	1012.0	5127	Fournier et al. 2010
Laboratory	Chlamydomonas reinhardtii	LabSeVI	7.7	0.8	1.5	1900	Fournier et al. 2010
Laboratory	Chlamydomonas reinhardtii	LabSeVI	7.7	7.9	6.0	760	Fournier et al. 2010
Laboratory	Chlamydomonas reinhardtii	LabSeVI	7.7	47.4	31.0	654	Fournier et al. 2010
Laboratory	Chlamydomonas reinhardtii	LabSeVI	7.7	197.4	123.8	627	Fournier et al. 2010
Laboratory	Chlamydomonas reinhardtii	LabSeVI	7.7	197.4	131.5	666	Geoffroy et al. 2007
Laboratory	Chlamydomonas reinhardtii	LabSeVI	7.7	394.8	345.9	876	Geoffroy et al. 2007
Laboratory	Chlamydomonas reinhardtii	LabSeVI	7.7	592.2	567.5	958	Geoffroy et al. 2007
Laboratory	Chlamydomonas reinhardtii	LabSeVI	0.0	5.0	224.9	45204	Ponton et al. 2018
Laboratory	Chlamydomonas reinhardtii	LabSeVI	0.0	5.0	215.6	43348	Ponton et al. 2018
Laboratory	Chlamydomonas reinhardtii	LabSeVI	0.0	5.0	154.7	31099	Ponton et al. 2018
Laboratory	Chlamydomonas reinhardtii	LabSeVI	0.5	5.0	28.6	5758	Ponton et al. 2018
Laboratory	Chlamydomonas reinhardtii	LabSeVI	2.4	5.0	4.8	970	Ponton et al. 2018
Laboratory	Chlamydomonas reinhardtii	LabSeVI	4.8	5.0	1.5	302	Ponton et al. 2018
Laboratory	Chlamydomonas reinhardtii	LabSeVI	9.6	5.0	1.4	275	Ponton et al. 2018
Laboratory	Chlamydomonas reinhardtii	LabSeVI	48.0	5.0	0.5	107	Ponton et al. 2018
Laboratory	Chlamydomonas reinhardtii	LabSeVI	192.1	5.0	0.2	47	Ponton et al. 2018
Laboratory	Chlamydomonas reinhardtii	LabSeVI	384.2	5.0	0.2	40	Ponton et al. 2018
Laboratory	Chlamydomonas reinhardtii	LabSeVI	14.4	5922.0	837.1	141	Vriens et al. 2018
Laboratory	Chlamydomonas reinhardtii	LabSeVI	28.8	5922.0	567.6	96	Vriens et al. 2018
Laboratory	Chlamydomonas reinhardtii	LabSeVI	72.0	5922.0	384.6	65	Vriens et al. 2018
Laboratory	Chrysophyceae, Dynophyceae, Euglenophyceae, bacteria	LabSeVI	0.0	5.0	36.5	7337	Ponton et al. 2018
Laboratory	Chrysophyceae, Dynophyceae, Euglenophyceae, bacteria	LabSeVI	0.0	5.0	17.2	3462	Ponton et al. 2018
Laboratory	Chrysophyceae, Dynophyceae, Euglenophyceae, bacteria	LabSeVI	0.2	5.0	13.3	2674	Ponton et al. 2018
Laboratory	Chrysophyceae, Dynophyceae, Euglenophyceae, bacteria	LabSeVI	2.2	5.0	14.0	2814	Ponton et al. 2018
Laboratory	Chrysophyceae, Dynophyceae, Euglenophyceae, bacteria	LabSeVI	28.8	5.0	7.1	1418	Ponton et al. 2018
Laboratory	Chrysophyceae, Dynophyceae, Euglenophyceae, bacteria	LabSeVI	288.2	5.0	5.7	1137	Ponton et al. 2018
Laboratory	Chlorophyta (natural periphyton)	LabSeVI	2.9	5.0	2.7	540	Markwart et al. 2018
Laboratory	Bacillariophyta (natural periphyton)	LabSeVI	3.8	5.0	3.2	640	Markwart et al. 2018
Laboratory	Chlorophyta/Bacillariophyta (natural periphyton)	LabSeVI	5.8	5.0	2.2	440	Markwart et al. 2018
Laboratory	Chlorophyta	LabSeVI	2.9	5.0	3.2	640	Markwart et al. 2018
Laboratory	Cyanophyta	LabSeVI	0.3	5.0	2.8	560	Markwart et al. 2018
Laboratory	Chlorophyta (natural periphyton)	LabSeVI	2.9	25.0	7.5	300	Markwart et al. 2018
Laboratory	Bacillariophyta (natural periphyton)	LabSeVI	3.8	25.0	8.6	344	Markwart et al. 2018
Laboratory	Chlorophyta/Bacillariophyta (natural periphyton)	LabSeVI	5.8	25.0	6.9	276	Markwart et al. 2018
Laboratory	Chlorophyta	LabSeVI	2.9	25.0	9.5	380	Markwart et al. 2018
Laboratory	Cyanophyta	LabSeVI	0.3	25.0	3.9	156	Markwart et al. 2018
Laboratory	P. subcapitata	LabSeVI	5.0	10.0	2.5	249	Lo et al. 2015
Laboratory	P. subcapitata	LabSeVI	5.0	18.0	3.4	190	Lo et al. 2015
Laboratory	P. subcapitata	LabSeVI	5.0	30.0	7.1	236	Lo et al. 2015
Laboratory	P. subcapitata	LabSeVI	5.0	54.0	13.3	246	Lo et al. 2015
Laboratory	P. subcapitata	LabSeVI	5.0	91.0	20.4	224	Lo et al. 2015
Laboratory	P. subcapitata	LabSeVI	155.0	9.0	1.0	109	Lo et al. 2015

Laboratory	P. subcapitata	LabSeVI	155.0	18.0	1.2	69	Lo et al. 2015
Laboratory	P. subcapitata	LabSeVI	155.0	31.0	2.1	67	Lo et al. 2015
Laboratory	P. subcapitata	LabSeVI	155.0	56.0	2.8	51	Lo et al. 2015
Laboratory	P. subcapitata	LabSeVI	155.0	97.0	4.6	48	Lo et al. 2015
Laboratory	P. subcapitata	LabSeVI	396.0	10.0	0.3	26	Lo et al. 2015
Laboratory	P. subcapitata	LabSeVI	396.0	18.0	0.3	19	Lo et al. 2015
Laboratory	P. subcapitata	LabSeVI	396.0	31.0	0.7	24	Lo et al. 2015
Laboratory	P. subcapitata	LabSeVI	396.0	55.0	1.8	32	Lo et al. 2015
Laboratory	P. subcapitata	LabSeVI	396.0	102.0	1.1	11	Lo et al. 2015
Laboratory	L. minor	LabSeVI	51.0	4.5	1.4	310	Lo et al. 2015
Laboratory	L. minor	LabSeVI	51.0	9.0	2.6	291	Lo et al. 2015
Laboratory	L. minor	LabSeVI	51.0	18.5	5.5	297	Lo et al. 2015
Laboratory	L. minor	LabSeVI	51.0	37.1	10.3	276	Lo et al. 2015
Laboratory	L. minor	LabSeVI	132.0	3.8	0.6	159	Lo et al. 2015
Laboratory	L. minor	LabSeVI	132.0	9.2	1.1	114	Lo et al. 2015
Laboratory	L. minor	LabSeVI	132.0	19.8	2.3	115	Lo et al. 2015
Laboratory	L. minor	LabSeVI	132.0	39.0	3.8	97	Lo et al. 2015
Laboratory	L. minor	LabSeVI	220.0	4.2	0.5	128	Lo et al. 2015
Laboratory	L. minor	LabSeVI	220.0	9.1	1.4	148	Lo et al. 2015
Laboratory	L. minor	LabSeVI	220.0	18.3	2.0	108	Lo et al. 2015
Laboratory	L. minor	LabSeVI	220.0	39.3	3.4	86	Lo et al. 2015
Laboratory	L. minor	LabSeVI	335.0	4.7	0.3	71	Lo et al. 2015
Laboratory	L. minor	LabSeVI	335.0	9.3	0.7	73	Lo et al. 2015
Laboratory	L. minor	LabSeVI	335.0	19.6	1.1	57	Lo et al. 2015
Laboratory	L. minor	LabSeVI	335.0	38.5	1.9	50	Lo et al. 2015
Laboratory	Ulva	LabSeVI	2958.6	197.4	0.6	3	Schiavon et al. 2012
Laboratory	Ulva	LabSeVI	2958.6	789.6	1.7	2	Schiavon et al. 2012
Laboratory	Ulva	LabSeVI	2958.6	3948.0	13.3	3	Schiavon et al. 2012
Laboratory	Ulva	LabSeVI	2958.6	5922.0	21.3	4	Schiavon et al. 2012
Laboratory	Ulva	LabSeVI	2958.6	7896.0	30.8	4	Schiavon et al. 2012
Laboratory	Selenastrum capricornutum	LabSeVI	3.3	10.0	17.0	1700	William et al. 1991
Laboratory	Selenastrum capricornutum	LabSeVI	3.3	100.0	156.0	1560	William et al. 1991
Laboratory	Selenastrum capricornutum	LabSeVI	33.0	10.0	4.0	400	William et al. 1991
Laboratory	Selenastrum capricornutum	LabSeVI	33.0	100.0	11.0	110	William et al. 1991
San Diego Creek constructed pond, California	Sediment+algae mean	Pond	NA	20.4	5.2	255	Presser and Luoma, 2009
Tulare Basin, evaporation ponds, California (range 109-500)	NA	Lentic	NA	25.6	9.9	388	Moore et al., 1990
Upper Newport Bay, California (range 101-776), in San Diego Creek	Suspended particulates	Pond	NA	1.9	0.3	148	Presser and Luoma, 2009
Benton Lake (pool 2), Montana	Sediment	Wetland	NA	10.4	3.5	337	Zhang and Moore, 1996
Kesterson Reservoir (SLD/pond 2), California (range 200-500)	sediment+organic detritus	Pond	NA	330.0	110.0	333	Presser and Piper, 1998
Benton Lake, Montana, pool 5	Sediment	Wetland	NA	0.7	0.4	473	Zhang and Moore, 1996
Benton Lake, Montana, pool 1 channel	Sediment	Wetland	NA	20.3	10.4	512	Zhang and Moore, 1996
Lower Great Lakes, Lake Ontario	NA	Lentic	NA	0.2	0.1	611	Ware, 2008
East Allen Reservoir, Wyoming	NA	Reservoir	NA	4.8	3.0	625	Birkner, 1978
Meeboer Lake, Wyoming	Sediment	Lentic	NA	0.3	0.2	667	Birkner, 1978
Diamond Lake, Wyoming	Sediment+Algae?	Lentic	NA	0.3	0.2	750	Birkner, 1978
Chevron Marsh (constructed), California (range 214-1241)	Sediment	Wetland	NA	7.4	4.4	595	Hansen and others, 1998
Miller's Lake, Colorado	NA	Lentic	NA	6.0	4.6	767	Birkner, 1978
San Diego Creek constructed marsh, California	Sediment+Algae mean	Wetland	NA	2.0	1.6	800	Presser and Luoma, 2009
Mac Mesa Reservoir, Colorado	Sediment	Reservoir	NA	2.2	1.8	818	Birkner, 1978
Sweitzer Lake, Colorado	Sediment + algae	Lentic	NA	9.4	9.1	968	Birkner, 1978
Desert Reservoir, Colorado	Sediment + algae	Reservoir	NA	12.5	12.1	968	Birkner, 1978
Salton Sea, California	NA	SalineLake	NA	0.9	1.1	1196	California Resources Agency, 2006
Twin Buttes Reservoir, Wyoming	Sediment + algae	Reservoir	NA	7.6	9.3	1224	Birkner, 1978
Galett Lake, Wyoming	Sediment + algae	Lentic	NA	0.8	1.1	1313	Birkner, 1978
Lower Great Lakes, Hamilton Harbor	NA	Lentic	NA	0.7	0.9	1388	Ware, 2008
Tulare Basin, evaporation ponds, California [b]	microphytes	Wetland	NA	8.8	11.8	1341	Fan et al., 2002
Cobb Lake, Colorado	Not sediment or algae?	Lentic	NA	3.8	6.0	1579	Birkner, 1978
Timber Lake, Colorado	Sediment?	Lentic	NA	2.1	1.3	619	Birkner, 1978
Larimer HWY 9 pond, Colorado	Sediment + algae	Pond	NA	15.9	27.3	1717	Birkner, 1978
Great Salt Lake, Utah	NA	SalineLake	NA	0.5	1.0	1759	Marden, 2008
Upper Mud River Reservoir at Palermo, West Virginia	NA	Reservoir	NA	3.7	6.7	1811	USGS, 2008
Wellington State Pond, Colorado	Sediment + algae	Pond	NA	1.7	3.3	1941	Birkner, 1978
Highline Reservoir, Colorado	Sediment + algae	Reservoir	NA	4.2	9.0	2143	Birkner, 1978
Belews Lake, North Carolina	NA	Reservoir	NA	10.9	30.5	2798	Cumbie, 1984; Lemly, 1985
Kesterson Reservoir (pond 8), California	Sediment, detritus, filamentous algae mean	Pond	NA	41.0	119.0	2902	Saiki and Lowe, 1987
Hyco Reservoir, North Carolina	NA	Reservoir	NA	11.5	27.0	2348	Bowie et al., 1996
Kesterson Reservoir (pond 11), California	NA	Pond	NA	9.0	32.0	3556	Saiki and Lowe, 1987
Great Marsh, Delaware	NA	Wetland	NA	0.0	0.5	14286	Velinsky and Cutter, 1991
Mesoscosms, Lake 114, ELA	periphyton	Lentic	NA	0.1	3.5	26769	Graves et al., 2019
Mesoscosms, Lake 114, ELA	periphyton	Lentic	NA	0.1	3.2	22587	Graves et al., 2019
Mesoscosms, Lake 114, ELA	periphyton	Lentic	NA	0.1	2.1	17087	Graves et al., 2019
Mesoscosms, Lake 114, ELA	periphyton	Lentic	NA	1.1	10.3	9097	Graves et al., 2019
Mesoscosms, Lake 114, ELA	periphyton	Lentic	NA	0.9	14.8	16109	Graves et al., 2019
Mesoscosms, Lake 114, ELA	periphyton	Lentic	NA	1.0	10.7	10888	Graves et al., 2019
Mesoscosms, Lake 114, ELA	periphyton	Lentic	NA	11.9	80.3	6750	Graves et al., 2019
Mesoscosms, Lake 114, ELA	periphyton	Lentic	NA	7.1	50.6	7144	Graves et al., 2019
Mesoscosms, Lake 114, ELA	periphyton	Lentic	NA	7.8	83.2	10610	Graves et al., 2019
Lake Arnoux, Quebec, Canada	Flocs	Lentic	NA	0.1	3.1	28972	Ponton and Hare, 2018_Flocs
Tilton Lake, Ontario, Canada	Flocs	Lentic	NA	0.2	6.2	37126	Ponton and Hare, 2018_Flocs
Lohi Lake, Ontario, Canada	Flocs	Lentic	NA	0.2	5.4	27692	Ponton and Hare, 2018_Flocs
Pine Lake, Ontario, Canada	Flocs	Lentic	NA	0.2	9.0	38462	Ponton and Hare, 2018_Flocs
Crooked Lake, Ontario, Canada	Flocs	Lentic	NA	0.3	6.2	21831	Ponton and Hare, 2018_Flocs
Silver Lake, Ontario, Canada	Flocs	Lentic	NA	0.3	5.2	15710	Ponton and Hare, 2018_Flocs
Lake Dufault, Quebec, Canada	Flocs	Lentic	NA	0.4	17.7	42530	Ponton and Hare, 2018_Flocs
Hannah Lake, Ontario, Canada	Flocs	Lentic	NA	0.5	13.9	28601	Ponton and Hare, 2018_Flocs
Lake Osisko, Quebec, Canada	Flocs	Lentic	NA	0.5	15.3	28281	Ponton and Hare, 2018_Flocs
Lake Pelletier, Quebec, Canada	Flocs	Lentic	NA	0.6	4.6	8062	Ponton and Hare, 2018_Flocs
Lake Rouyn, Quebec, Canada	Flocs	Lentic	NA	1.4	28.5	19792	Ponton and Hare, 2018_Flocs
Kelly Lake, Ontario, Canada	Flocs	Lentic	NA	3.1	28.0	9136	Ponton and Hare, 2018_Flocs
Lake	Sediment	Lentic	NA	0.1	5.7	57000	Muscatello et al. 2008
Lake	Sediment	Lentic	NA	0.7	25.6	36571	Muscatello et al. 2008
Lake	Sediment	Lentic	NA	2.7	62.2	23037	Muscatello et al. 2008
Lake	periphyton	Lentic	NA	0.1	0.3	2900	Muscatello et al. 2008
Lake	periphyton	Lentic	NA	0.7	1.0	1443	Muscatello et al. 2008
Lake	periphyton	Lentic	NA	2.7	3.8	1389	Muscatello et al. 2008
lake	periphyton	Lentic	NA	0.3	0.5	1733	Mailman Thesis
lake	periphyton	Lentic	NA	0.4	0.9	2514	Mailman Thesis
lake	periphyton	Lentic	NA	0.7	0.9	1314	Mailman Thesis
lake	periphyton	Lentic	NA	0.6	2.9	4867	Mailman Thesis
lake	periphyton	Lentic	NA	0.8	1.8	2250	Mailman Thesis
lake	periphyton	Lentic	NA	1.1	4.1	3700	Mailman Thesis
lake	Sediment	Lentic	NA	0.3	7.5	25000	Mailman Thesis
lake	Sediment	Lentic	NA	0.4	5.0	14286	Mailman Thesis
lake	Sediment	Lentic	NA	0.7	7.5	10714	Mailman Thesis
lake	Sediment	Lentic	NA	0.6	7.6	12667	Mailman Thesis
lake	Sediment	Lentic	NA	0.8	15.0	18750	Mailman Thesis
lake	Sediment	Lentic	NA	1.1	10.0	9091	Mailman Thesis
Elk Valkey - Lentic	periphyton	Lentic	NA	41.9	43.4	1036	Orr et al. 2012 - Many sources
Elk Valkey - Lentic	periphyton	Lentic	NA	30.3	8.2	270	Orr et al. 2012 - Many sources
Elk Valkey - Lentic	periphyton	Lentic	NA	0.4	1.0	2400	Orr et al. 2012 - Many sources
Elk Valkey - Lentic	periphyton	Lentic	NA	0.4	0.4	875	Orr et al. 2012 - Many sources
Elk Valkey - Lentic	periphyton	Lentic	NA	0.2	1.0	4890	Orr et al. 2012 - Many sources
Elk Valkey - Lentic	periphyton	Lentic	NA	0.2	1.9	9300	Orr et al. 2012 - Many sources
Elk Valkey - Lentic	periphyton	Lentic	NA	64.5	83.6	1296	Orr et al. 2012 - Many sources
Elk Valkey - Lentic	periphyton	Lentic	NA	35.3	51.0	1446	Orr et al. 2012 - Many sources
Elk Valkey - Lentic	periphyton	Lentic	NA	11.6	14.6	1259	Orr et al. 2012 - Many sources
Elk Valkey - Lentic	periphyton	Lentic	NA	16.3	18.5	1135	Orr et al. 2012 - Many sources

Elk Valkey - Lentic	periphyton	Lentic	NA	18.0	18.1	1008	Orr et al. 2012 - Many sources
Elk Valkey - Lentic	periphyton	Lentic	NA	14.0	1.1	81	Orr et al. 2012 - Many sources
Elk Valkey - Lentic	periphyton	Lentic	NA	3.2	7.5	2335	Orr et al. 2012 - Many sources
Elk Valkey - Lentic	periphyton	Lentic	NA	0.2	3.3	16470	Orr et al. 2012 - Many sources
Jordan river - lentic (pond)	Sediment	Lentic	NA	2.9	5.0	1724	Hillwalker et al. 2006
Jordan river - lentic (pond)	Sediment	Lentic	NA	6.3	2.1	333	Hillwalker et al. 2006
Jordan river - lentic (pond)	Sediment	Lentic	NA	1.9	3.8	2000	Hillwalker et al. 2006
Jordan river - lentic (pond)	Sediment	Lentic	NA	5.0	2.8	560	Hillwalker et al. 2006
Elk valley - lentic	Sediment	Lentic	NA	7.2	7.9	1105	Orr et al. 2006
Elk valley - lentic	Sediment	Lentic	NA	46.0	2.8	61	Orr et al. 2006
Elk valley - lentic	Sediment	Lentic	NA	88.0	26.0	295	Orr et al. 2006
Elk valley - lentic	Sediment	Lentic	NA	0.7	3.0	4286	Orr et al. 2006
Elk valley - lentic	Sediment	Lentic	NA	0.5	2.0	4000	Orr et al. 2006
Elk valley - lentic	Sediment	Lentic	NA	1.2	3.0	2609	Orr et al. 2006
Elk valley - lentic	Flocs	Lentic	NA	7.2	5.6	776	Orr et al. 2006
Elk valley - lentic	Flocs	Lentic	NA	46.0	5.5	119	Orr et al. 2006
Elk valley - lentic	Flocs	Lentic	NA	88.0	3.2	36	Orr et al. 2006
Elk valley - lentic	Flocs	Lentic	NA	0.5	4.4	8800	Orr et al. 2006
Mud Slough, CA (GT5; Fall 1987)	Detritus	Creek	NA	3.0	22.0	7333	Saiki et al. 1993
Mud Slough, CA (GT5; Fall 1987)	Algae	Creek	NA	3.0	7.4	2467	Saiki et al. 1993
Mud Slough, CA (GT5; Spring 1987)	Detritus	Creek	NA	9.0	7.9	878	Saiki et al. 1993
Mud Slough, CA (GT5; Spring 1987)	Algae	Creek	NA	9.0	1.6	178	Saiki et al. 1993
Salt Slough, CA (GT4; Fall 1987)	Detritus	Creek	NA	3.0	8.9	2967	Saiki et al. 1993
Salt Slough, CA (GT4; Fall 1987)	Algae	Creek	NA	3.0	0.4	127	Saiki et al. 1993
Salt Slough, CA (GT4; Spring 1987)	Detritus	Creek	NA	13.0	7.9	608	Saiki et al. 1993
Salt Slough, CA (GT4; Spring 1987)	Algae	Creek	NA	13.0	2.4	185	Saiki et al. 1993
Lower San Diego Creek, CA	Algae	Creek	NA	4.1	0.6	156	Presser and Luoma, 2009
Fording River, BC	Fine silt, decomposing Hydrurus	River	NA	8.6	2.4	280	McDonald and Stosher 1998
Fording River, BC	Fine silt	River	NA	9.6	1.5	159	McDonald and Stosher 1998
Michel Creek, BC	Silt, fine sand	Creek	NA	0.6	0.5	833	McDonald and Stosher 1998
Michel Creek, BC	Silt, decomposing Hydrurus	Creek	NA	7.1	2.3	327	McDonald and Stosher 1998
Merced River, CA (ET6; Fall 1987)	Detritus	River	NA	0.5	1.2	2400	Saiki et al. 1993
Merced River, CA (ET6; Fall 1987)	Algae	River	NA	0.5	1.9	3800	Saiki et al. 1993
Merced River, CA (ET6; Spring 1987)	Detritus	River	NA	0.5	1.1	2200	Saiki et al. 1993
Merced River, CA (ET6; Spring 1987)	Algae	River	NA	0.5	0.2	300	Saiki et al. 1993
San Joaquin River, CA (SJR1; Fall 1987)	Detritus	River	NA	0.5	0.4	840	Saiki et al. 1993
San Joaquin River, CA (SJR1; Fall 1987)	Algae	River	NA	0.5	0.3	680	Saiki et al. 1993
San Joaquin River, CA (SJR1; Spring 1987)	Detritus	River	NA	0.5	0.6	1160	Saiki et al. 1993
San Joaquin River, CA (SJR1; Spring 1987)	Algae	River	NA	0.5	0.1	200	Saiki et al. 1993
San Joaquin River, CA (SJR2; Fall 1987)	Detritus	River	NA	3.0	6.6	2200	Saiki et al. 1993
San Joaquin River, CA (SJR2; Fall 1987)	Algae	River	NA	3.0	1.2	400	Saiki et al. 1993
San Joaquin River, CA (SJR2; Spring 1987)	Detritus	River	NA	11.0	3.4	309	Saiki et al. 1993
San Joaquin River, CA (SJR2; Spring 1987)	Algae	River	NA	11.0	1.3	118	Saiki et al. 1993
San Joaquin River, CA (SJR3; Fall 1987)	Detritus	River	NA	1.0	1.2	1200	Saiki et al. 1993
San Joaquin River, CA (SJR3; Fall 1987)	Algae	River	NA	1.0	0.4	390	Saiki et al. 1993
San Joaquin River, CA (SJR3; Spring 1987)	Detritus	River	NA	1.0	1.3	1300	Saiki et al. 1993
San Joaquin River, CA (SJR3; Spring 1987)	Algae	River	NA	1.0	0.5	500	Saiki et al. 1993
Stanislaus River, CA (ET7; Fall 1987)	Detritus	River	NA	0.5	0.8	1600	Saiki et al. 1993
Stanislaus River, CA (ET7; Fall 1987)	Algae	River	NA	0.5	0.1	140	Saiki et al. 1993
Stanislaus River, CA (ET7; Spring 1987)	Detritus	River	NA	0.5	0.7	1420	Saiki et al. 1993
Stanislaus River, CA (ET7; Spring 1987)	Algae	River	NA	0.5	0.2	480	Saiki et al. 1993
Luscar Creek, AB, Coal Mining Creek	Biofilm, filamentous algae, fine sed.	Creek	NA	10.7	3.7	346	Casey 2005
Deerlick Creek, AB, Coal Mining Creek	Biofilm, fine sed.	Creek	NA	0.2	1.0	5000	Casey 2005
Elk River, BC Coal Mining River	Silt, sand	River	NA	2.2	1.2	536	McDonald and Stosher 1998
Angus Creek, ID	Aquatic plant	Creek	NA	0.3	0.9	3600	Hamilton et al. 2002
Angus Creek, ID	Aquatic plant	Creek	NA	6.0	2.0	333	Hamilton and Buhl 2003a
Angus Creek, ID	Aquatic plant	Creek	NA	1.0	2.8	2800	Hamilton and Buhl 2003b
Angus Creek, ID	Aquatic plant	Creek	NA	0.8	1.1	1348	GYC 2005
Trail Creek, ID	Aquatic plant	Creek	NA	0.3	0.8	3200	Hamilton et al. 2002
Trail Creek, ID	Aquatic plant	Creek	NA	5.0	1.7	340	Hamilton and Buhl 2003a
Upper Slug Creek, ID	Aquatic plant	Creek	NA	0.3	1.1	4400	Hamilton et al. 2002
Upper Slug Creek, ID	Aquatic plant	Creek	NA	7.0	1.6	229	Hamilton and Buhl 2003a
Lower Slug Creek, ID	Aquatic plant	Creek	NA	0.3	1.5	6000	Hamilton et al. 2002
Lower Slug Creek, ID	Aquatic plant	Creek	NA	6.0	1.7	283	Hamilton and Buhl 2003a
Dry Valley Creek, ID	Aquatic plant	Creek	NA	0.3	3.8	15200	Hamilton et al. 2002
Dry Valley Creek, ID	Aquatic plant	Creek	NA	8.0	4.4	550	Hamilton and Buhl 2003a
Sheep Creek, ID	Aquatic plant	Creek	NA	0.3	0.6	2400	Hamilton et al. 2002
Sheep Creek, ID	Aquatic plant	Creek	NA	8.0	1.2	150	Hamilton and Buhl 2003a
Upper Georgetown Creek, ID	Aquatic plant	Creek	NA	11.0	3.7	336	Hamilton and Buhl 2003b
Upper East Mill Creek, ID	Aquatic plant	Creek	NA	70.0	30.3	433	Hamilton and Buhl 2003a; TtEMI 2002
Lower East Mill Creek, ID	Aquatic plant	Creek	NA	69.0	49.9	723	Hamilton and Buhl 2003a; TtEMI 2002
Smoky Creek, ID	Aquatic plant	Creek	NA	0.3	0.8	3160	GYC 2005
Deer Creek, ID	Aquatic plant	Creek	NA	1.6	3.6	2293	GYC 2005
Crow Creek above Sage Creek, ID	Aquatic plant	Creek	NA	1.0	1.8	1846	GYC 2005
Crow Creek on Toner Ranch, ID	Aquatic plant	Creek	NA	3.6	2.3	651	GYC 2005
Sage Creek on Porter Ranch, ID	Aquatic plant	Creek	NA	7.7	3.9	501	GYC 2005
Alexander Creek, BC (AC)	Cladophora	Creek	NA	0.9	4.5	4989	Orr et al. 2006
Alexander Creek, BC (AC)	Epilithon/periphyton	Creek	NA	0.6	0.8	1333	Orr et al. 2012
Fording River, BC (FR)	Epilithon (biofilm)[2]	River	NA	20.1	5.4	270	Orr et al. 2006
Fording River, BC (FR)	Cladophora	River	NA	20.1	1.1	55	Orr et al. 2006
Fording River, BC (FR)	Moss	River	NA	20.1	7.9	395	Orr et al. 2006
Line Creek, BC (LC)	Epilithon (biofilm)[2]	Creek	NA	20.9	2.2	105	Orr et al. 2006
Elk River, BC (EL1 [747])	Epilithon/periphyton	River	NA	2.2	1.3	582	Orr et al. 2012
Elk River, BC (EL1 [L11])	Epilithon/periphyton	River	NA	3.3	1.5	455	Orr et al. 2012
Elk River, BC (EL1)	Epilithon/periphyton	River	NA	8.6	2.3	267	Orr et al. 2012
Elk River, BC (EL1)	Epilithon/periphyton	River	NA	4.2	2.6	619	Orr et al. 2012
Elk River, BC (EL12 [745])	Epilithon/periphyton	River	NA	0.1	0.3	3100	Orr et al. 2012
Elk River, BC (EL12 [L1])	Epilithon/periphyton	River	NA	0.8	1.3	1625	Orr et al. 2012
Elk River, BC (EL12)	Epilithon/periphyton	River	NA	0.7	2.7	3857	Orr et al. 2012
Elk River, BC (EL20 [750])	Epilithon/periphyton	River	NA	0.4	0.8	1950	Orr et al. 2012
Elk River, BC (EL20 [L4])	Epilithon/periphyton	River	NA	0.9	0.7	778	Orr et al. 2012
Ewin Creek, BC (L17)	Epilithon/periphyton	Creek	NA	1.3	2.0	1538	Orr et al. 2012
Fording River, BC (FO22 [746])	Epilithon/periphyton	River	NA	8.6	1.6	181	Orr et al. 2012
Fording River, BC (FO22 [L3])	Epilithon/periphyton	River	NA	17.0	1.9	112	Orr et al. 2012
Fording River, BC (FO23)	Epilithon/periphyton	River	NA	37.3	8.9	239	Orr et al. 2012
Fording River, BC (FO23)	Epilithon/periphyton	River	NA	23.9	3.8	159	Orr et al. 2012
Fording River, BC (FO9 [L5])	Epilithon/periphyton	River	NA	8.3	1.3	157	Orr et al. 2012
Fording River, BC (MP1)	Epilithon/periphyton	River	NA	12.1	5.1	421	Orr et al. 2012
Line Creek, BC (LI24 [L6])	Epilithon/periphyton	Creek	NA	1.8	2.6	1444	Orr et al. 2012
Line Creek, BC (LI8 [749])	Epilithon/periphyton	Creek	NA	10.5	1.3	122	Orr et al. 2012
Line Creek, BC (LI8)	Epilithon/periphyton	Creek	NA	24.5	4.2	171	Orr et al. 2012
Michel Creek, BC (MI2 [751])	Epilithon/periphyton	Creek	NA	7.1	1.3	177	Orr et al. 2012
Michel Creek, BC (MI2 [L9])	Epilithon/periphyton	Creek	NA	7.4	1.5	203	Orr et al. 2012
Michel Creek, BC (MI2)	Epilithon/periphyton	Creek	NA	7.4	3.3	446	Orr et al. 2012
Michel Creek, BC (MI2)	Epilithon/periphyton	Creek	NA	6.1	2.1	344	Orr et al. 2012
Michel Creek, BC (MI3)	Epilithon/periphyton	Creek	NA	1.9	1.3	684	Orr et al. 2012
Cheviot Creek, AB (CH2)	Periphyton	Creek	NA	32.0	1.6	49	Golder 2013a; Teck Coal 2013
Gregg River, AB (GR1)	Periphyton	River	NA	0.8	0.9	1190	Golder 2013a; Teck Coal 2013
Gregg River, AB (GR2)	Periphyton	River	NA	13.9	1.3	95	Golder 2013a; Teck Coal 2013
Gregg River, AB (GR2)	Periphyton	River	NA	4.0	1.8	448	Golder 2011; Teck Coal 2013
Gregg River, AB (GR5)	Periphyton	River	NA	4.4	1.3	305	Golder 2011; Teck Coal 2013
Gregg River, AB (GR6)	Periphyton	River	NA	3.4	0.8	226	Golder 2013a; Teck Coal 2013
Gregg River, AB (GR6)	Periphyton	River	NA	1.7	1.1	653	Golder 2011; Teck Coal 2013
Harris Creek, AB (HC2)	Periphyton	Creek	NA	0.5	0.4	722	Golder 2013a; Teck Coal 2013
Luscar Creek, AB (LUS1)	Periphyton	Creek	NA	1.2	2.3	1892	Golder 2013a; Teck Coal 2013
Luscar Creek, AB (LUS1)	Periphyton	Creek	NA	1.7	1.6	953	Golder 2011; Teck Coal 2013
Luscar Creek, AB (LUS2)	Periphyton	Creek	NA	14.7	2.1	140	Golder 2013a; Teck Coal 2013

Luscar Creek, AB (LUS2)	Periphyton	Creek	NA	11.2	1.1	102	Golder 2011; Teck Coal 2013
Luscar Creek, AB (LUS4)	Periphyton	Creek	NA	18.8	3.2	168	Golder 2011; Teck Coal 2013
Mackenzie Creek, AB (MK1)	Periphyton	Creek	NA	0.4	1.0	2375	Golder 2011; Teck Coal 2013
Mary Gregg Creek, AB (MG2)	Periphyton	Creek	NA	17.4	1.8	105	Golder 2013a; Teck Coal 2013
Mary Gregg Creek, AB (MG2)	Periphyton	Creek	NA	7.5	3.8	504	Golder 2011; Teck Coal 2013
Mary Gregg Creek, AB (MG3)	Periphyton	Creek	NA	3.1	1.8	577	Golder 2013a; Teck Coal 2013
McLeod River, AB (MR1)	Periphyton	River	NA	0.3	2.2	8462	Golder 2013a; Teck Coal 2013
McLeod River, AB (MR1)	Periphyton	River	NA	0.3	0.7	2300	Golder 2011; Teck Coal 2013
McLeod River, AB (MR2)	Periphyton	River	NA	3.0	1.1	370	Golder 2013a; Teck Coal 2013
McLeod River, AB (MR2)	Periphyton	River	NA	2.5	1.5	584	Golder 2011; Teck Coal 2013
McLeod River, AB (MR3)	Periphyton	River	NA	1.6	2.2	1350	Golder 2013a; Teck Coal 2013
McLeod River, AB (MR4)	Periphyton	River	NA	1.6	1.4	844	Golder 2013a; Teck Coal 2013
McLeod River, AB (MR4)	Periphyton	River	NA	1.0	0.6	590	Golder 2011; Teck Coal 2013
McLeod River, AB (MR5)	Periphyton	River	NA	0.8	0.8	938	Golder 2013a; Teck Coal 2013
McLeod River, AB (MR6)	Periphyton	River	NA	2.5	0.8	332	Golder 2013a; Teck Coal 2013
McLeod River, AB (MR6)	Periphyton	River	NA	2.0	1.1	550	Golder 2011; Teck Coal 2013
McLeod River, AB (MR7)	Periphyton	River	NA	1.2	0.7	600	Golder 2013a; Teck Coal 2013
McLeod River, AB (MR7)	Periphyton	River	NA	1.3	0.4	277	Golder 2011; Teck Coal 2013
Prospect Creek, AB (PC1)	Periphyton	Creek	NA	1.9	1.2	616	Golder 2013a; Teck Coal 2013
Prospect Creek, AB (PC3)	Periphyton	Creek	NA	1.6	0.7	444	Golder 2013a; Teck Coal 2013
Prospect Creek, AB (PC3)	Periphyton	Creek	NA	0.7	0.4	629	Golder 2011; Teck Coal 2013
Thornton Creek, AB (TH1)	Periphyton	Creek	NA	0.5	2.2	4440	Golder 2013a; Teck Coal 2013
Thornton Creek, AB (TH2)	Periphyton	Creek	NA	4.3	5.5	1288	Golder 2013a; Teck Coal 2013
SF Tincup Creek (SFTC-1)	Periphyton	Creek	NA	0.4	0.8	1907	Formation Environmental and HabiTech 2012
SF Tincup Creek (SFTC-1)	Periphyton	Creek	NA	0.2	0.6	3150	Formation Environmental and HabiTech 2012
SF Tincup Creek (SFTC-1)	Periphyton	Creek	NA	0.4	1.2	2614	Formation Environmental and HabiTech 2012
SF Tincup Creek (SFTC-1)	Periphyton	Creek	NA	0.7	0.2	224	Formation Environmental and HabiTech 2012
Crow Creek, ID (CC-75)	Periphyton	Creek	NA	0.6	1.0	1772	Formation Environmental and HabiTech 2012
Crow Creek, ID (CC-75)	Periphyton	Creek	NA	0.5	0.7	1478	Formation Environmental and HabiTech 2012
Crow Creek, ID (CC-75)	Periphyton	Creek	NA	0.3	1.1	3333	Formation Environmental and HabiTech 2012
Crow Creek, ID (CC-75)	Periphyton	Creek	NA	1.2	2.7	2250	Formation Environmental and HabiTech 2012
Crow Creek, ID (CC-75)	Periphyton	Creek	NA	0.8	0.6	688	Formation Environmental and HabiTech 2012
Crow Creek, ID (CC-150)	Periphyton	Creek	NA	0.7	1.2	1791	Formation Environmental and HabiTech 2012
Crow Creek, ID (CC-150)	Periphyton	Creek	NA	0.9	1.4	1489	Formation Environmental and HabiTech 2012
Crow Creek, ID (CC-150)	Sediment	Creek	NA	0.7	0.8	1132	Formation Environmental and HabiTech 2012
Crow Creek, ID (CC-150)	Periphyton	Creek	NA	1.4	2.4	1714	Formation Environmental and HabiTech 2012
Crow Creek, ID (CC-150)	Periphyton	Creek	NA	1.6	0.7	406	Formation Environmental and HabiTech 2012
Crow Creek, ID (CC-350)	Periphyton	Creek	NA	0.8	1.5	1829	Formation Environmental and HabiTech 2012
Crow Creek, ID (CC-350)	Periphyton	Creek	NA	1.1	3.3	3000	Formation Environmental and HabiTech 2012
Crow Creek, ID (CC-350)	Periphyton	Creek	NA	0.3	0.8	2962	Formation Environmental and HabiTech 2012
Crow Creek, ID (CC-350)	Periphyton	Creek	NA	0.9	3.4	3820	Formation Environmental and HabiTech 2012
Crow Creek, ID (CC-350)	Periphyton	Creek	NA	1.3	0.6	454	Formation Environmental and HabiTech 2012
Deer Creek, ID (DC-600)	Periphyton	Creek	NA	1.3	1.2	923	Formation Environmental and HabiTech 2012
Deer Creek, ID (DC-600)	Periphyton	Creek	NA	1.5	7.4	4960	Formation Environmental and HabiTech 2012
Deer Creek, ID (DC-600)	Periphyton	Creek	NA	1.4	8.7	6214	Formation Environmental and HabiTech 2012
Deer Creek, ID (DC-600)	Periphyton	Creek	NA	3.4	1.7	485	Formation Environmental and HabiTech 2012
Hoopes Spring, ID (HS)	Periphyton	Creek	NA	17.4	2.2	126	Formation Environmental and HabiTech 2012
Hoopes Spring, ID (HS)	Periphyton	Creek	NA	20.5	12.0	585	Formation Environmental and HabiTech 2012
Hoopes Spring, ID (HS)	Periphyton	Creek	NA	21.4	3.9	182	Formation Environmental and HabiTech 2012
Hoopes Spring, ID (HS)	Periphyton	Creek	NA	27.3	15.0	549	Formation Environmental and HabiTech 2012
Hoopes Spring, ID (HS)	Periphyton	Creek	NA	53.6	35.2	657	Formation Environmental and HabiTech 2012
Hoopes Spring, ID (HS-3)	Periphyton	Creek	NA	9.2	6.5	707	Formation Environmental and HabiTech 2012
Hoopes Spring, ID (HS-3)	Periphyton	Creek	NA	18.0	12.0	667	Formation Environmental and HabiTech 2012
Hoopes Spring, ID (HS-3)	Periphyton	Creek	NA	16.1	6.2	385	Formation Environmental and HabiTech 2012
Hoopes Spring, ID (HS-3)	Periphyton	Creek	NA	26.0	28.5	1096	Formation Environmental and HabiTech 2012
Hoopes Spring, ID (HS-3)	Periphyton	Creek	NA	37.5	24.2	645	Formation Environmental and HabiTech 2012
Sage Creek, ID (LSV-2C)	Periphyton	Creek	NA	9.3	2.6	280	Formation Environmental and HabiTech 2012
Sage Creek, ID (LSV-2C)	Periphyton	Creek	NA	13.5	8.1	599	Formation Environmental and HabiTech 2012
Sage Creek, ID (LSV-2C)	Periphyton	Creek	NA	14.3	18.5	1294	Formation Environmental and HabiTech 2012
Sage Creek, ID (LSV-2C)	Periphyton	Creek	NA	14.1	11.6	823	Formation Environmental and HabiTech 2012
Sage Creek, ID (LSV-2C)	Periphyton	Creek	NA	23.4	4.4	187	Formation Environmental and HabiTech 2012
Sage Creek, ID (LSV-4)	Periphyton	Creek	NA	6.8	7.4	1091	Formation Environmental and HabiTech 2012
Sage Creek, ID (LSV-4)	Periphyton	Creek	NA	10.1	11.7	1158	Formation Environmental and HabiTech 2012
Crow Creek, ID (CC-1A)	Periphyton	Creek	NA	2.7	3.6	1348	Formation Environmental and HabiTech 2012
Crow Creek, ID (CC-1A)	Periphyton	Creek	NA	1.2	3.4	2825	Formation Environmental and HabiTech 2012
Crow Creek, ID (CC-1A)	Periphyton	Creek	NA	2.2	3.2	1455	Formation Environmental and HabiTech 2012
Crow Creek, ID (CC-1A)	Periphyton	Creek	NA	2.9	7.1	2448	Formation Environmental and HabiTech 2012
Crow Creek, ID (CC-1A)	Periphyton	Creek	NA	6.7	5.9	875	Formation Environmental and HabiTech 2012
Crow Creek, ID (CC-3A)	Periphyton	Creek	NA	2.9	3.1	1069	Formation Environmental and HabiTech 2012
Crow Creek, ID (CC-3A)	Periphyton	Creek	NA	1.4	1.9	1350	Formation Environmental and HabiTech 2012
Crow Creek, ID (CC-3A)	Periphyton	Creek	NA	1.8	3.8	2111	Formation Environmental and HabiTech 2012
Crow Creek, ID (CC-3A)	Periphyton	Creek	NA	2.6	14.9	5731	Formation Environmental and HabiTech 2012
Crow Creek, ID (CC-3A)	Periphyton	Creek	NA	5.8	1.7	288	Formation Environmental and HabiTech 2012
San Diego Creek, California, urban	sediment+algae average	Creek	NA	14.4	1.5	107	Presser and Luoma, 2009
Alamo River, California, agricultural	Suspended particulates	Creek	NA	6.4	0.7	110	LeBlanc and Schroeder, 2008
Fording River, British Columbia, Canada, coal	Sediment	Creek	NA	18.0	2.2	122	Harding et al., 2005
New River, California, agricultural	Suspended particulates	Creek	NA	3.9	1.0	256	LeBlanc and Schroeder, 2008
Sage Creek, Idaho	NA	Creek	NA	7.7	3.8	494	Greater Yellowstone Coalition, 2005; 2006
Crow Creek at Toner, Idaho	NA	Creek	NA	3.5	2.3	657	Greater Yellowstone Coalition, 2005; 2006
Mud River at Spurlock, West Virginia	NA	River	NA	6.3	6.9	1104	USGS, 2008
Angus Creek, Idaho	Macrophytes	Creek	NA	0.8	1.1	1341	Greater Yellowstone Coalition, 2005; 2006;
Big Canyon Wash (sites 1 and 2), California	NA	Creek	NA	21.5	32.2	1498	Presser and Luoma, 2009
Crow Creek above Sage Creek, Idaho	NA	Creek	NA	1.0	1.8	1818	Greater Yellowstone Coalition, 2005; 2006
Thompson Creek, Idaho	NA	Creek	NA	1.8	3.4	1943	GEI Consultants Inc., 2008
Deer Creek, Idaho	NA	Creek	NA	1.6	3.6	2250	Greater Yellowstone Coalition, 2005; 2006
Big Canyon Wash (site 3) California	NA	Creek	NA	24.0	75.6	3150	Presser and Luoma, 2009
Delaware River (tidal freshwater), Delaware	NA	River	NA	0.3	1.1	4038	Riedel and Sanders, 1998
Mud River, West Virginia	biofilm (unfractionated)	River	NA	0.1	1.3	13000	Arnold et al. 2017
Mud River, West Virginia	biofilm (unfractionated)	River	NA	6.1	2.7	443	Arnold et al. 2017
Mud River, West Virginia	diatom/sediment fraction	River	NA	6.1	9.1	1492	Arnold et al. 2017
Mud River, West Virginia	filamentous green algae fraction	River	NA	6.1	0.5	84	Arnold et al. 2017
Jordan River - lotic (creek)	Sediment	Creek	NA	1.8	0.8	444	Hillwalker et al. 2006
Jordan River - lotic (creek)	Sediment	Creek	NA	2.8	1.3	464	Hillwalker et al. 2006
Jordan River - lotic (creek)	Sediment	Creek	NA	1.6	1.6	1000	Hillwalker et al. 2006
Jordan River - lotic (creek)	Sediment	Creek	NA	4.0	0.8	200	Hillwalker et al. 2006
Elk Valley - lotic	Sediment	River	NA	0.9	0.9	1000	Orr et al. 2006

Table S2: Water total dissolved selenium concentrations and speciation

Country	Region	Type	Site	Total Se (ug/L)	Se(IV) (ug/L)	Se(VI) (ug/L)	OrgSe (ug/L)	SO4 (mg/L)	Reference
USA	New York	Sea	Jamaica Bay	0.14					Bakker et al. 2016
USA	New York	Sea	West Jones Beach	0.11					Bakker et al. 2016
USA	New York	Sea	Manhasset Bay	0.15					Bakker et al. 2016
USA	New York	Sea	Oyster Bay	0.16					Bakker et al. 2016
USA	New York	Sea	Westhampton Bay	0.17					Bakker et al. 2016
Chine	Tianjin	Sea	Bohai Bay		0.72	0.36			Duan et al. 2010
Russie	Karabash	Lentic	Serebry Lake	0.60					Gashkina et al. 2015
Russie	Karabash	Lentic	Seliger Lake	0.15					Gashkina et al. 2015
Turquie	Sivas	Lotic	Kilizirmak River, Sivas		1.43	2.70			Gurkan et al. 2011
Turquie	Sivas	Lentic	Hafik Lake		0.10	0.10			Gurkan et al. 2011
USA	Florida	Sea	Sarasota Bay, Florida	0.42					Hong et al. 2011
Canada	Bristish Columbia	Lentic	End-pit lake Coal	28.00				365	Luek and Rassmussen, 2017
Slovenia	Notranjska	Lotic	REF	0.07					Mechora et al. 2014
Slovenia	Notranjska	Lotic	Lipzenjscica	0.14					Mechora et al. 2014
Slovenia	Notranjska	Lotic	Zerovniscica Z1	0.22					Mechora et al. 2014
Slovenia	Notranjska	Lotic	Z2	0.12					Mechora et al. 2014
Slovenia	Notranjska	Lotic	Z3	0.23					Mechora et al. 2014
Slovenia	Notranjska	Lotic	Z4	0.21					Mechora et al. 2014
Slovenia	Notranjska	Lotic	Z5	0.17					Mechora et al. 2014
Slovenia	Notranjska	Lotic	Izica	0.40					Mechora et al. 2014
Slovenia	Notranjska	Lotic	Psata P1	0.17					Mechora et al. 2014
Slovenia	Notranjska	Lotic	P2	0.27					Mechora et al. 2014
Slovenia	Notranjska	Lotic	Ihan	0.06					Mechora et al. 2014
Canada	Quebec	Lentic	AR-2	0.06	0.02	0.03	0.01		Ponton and Hare, 2013
Canada	Quebec	Lentic	Duprat_10	0.10	0.04	0.01	0.05		Ponton and Hare, 2013
Canada	Quebec	Lentic	AR-4_11	0.11	0.01	0.09	0.01		Ponton and Hare, 2013
Canada	Quebec	Lentic	DAS-8_11	0.12	0.01	0.07	0.04		Ponton and Hare, 2013
Canada	Ontario	Lentic	Tilton_11	0.17	0.01	0.02	0.14		Ponton and Hare, 2013
Canada	Ontario	Lentic	Lohi_11	0.20	0.01	0.03	0.16		Ponton and Hare, 2013
Canada	Ontario	Lentic	Mc Farlane_10	0.23	0.15	0.01	0.07		Ponton and Hare, 2013
Canada	Ontario	Lentic	Pine_11	0.24	0.01	0.14	0.08		Ponton and Hare, 2013
Canada	Ontario	Lentic	Crooked_11	0.29	0.01	0.03	0.24		Ponton and Hare, 2013
Canada	Ontario	Lentic	Silver_11	0.33	0.03	0.03	0.27		Ponton and Hare, 2013
Canada	Ontario	Lentic	Raft_10	0.33	0.07	0.08	0.19		Ponton and Hare, 2013
Canada	Quebec	Lentic	Dufault_10	0.42	0.17	0.01	0.23		Ponton and Hare, 2013
Canada	Ontario	Lentic	Crooked_10	0.49	0.06	0.01	0.42		Ponton and Hare, 2013
Canada	Quebec	Lentic	Dufault_11	0.45	0.12	0.04	0.28		Ponton and Hare, 2013
Canada	Ontario	Lentic	Hannah_11	0.50	0.14	0.04	0.31		Ponton and Hare, 2013
Canada	Ontario	Lentic	Hannah_10	0.54	0.25	0.01	0.28		Ponton and Hare, 2013
Canada	Quebec	Lentic	Osisko_10	0.54	0.31	0.08	0.15		Ponton and Hare, 2013
Canada	Quebec	Lentic	Osisko_11	0.56	0.20	0.15	0.21		Ponton and Hare, 2013
Canada	Quebec	Lentic	Pelletier_11	0.57	0.25	0.07	0.25		Ponton and Hare, 2013
Canada	Quebec	Lentic	Pelletier_10	0.60	0.28	0.08	0.24		Ponton and Hare, 2013
Canada	Quebec	Lentic	Rouyn_11	1.44	0.88	0.32	0.24		Ponton and Hare, 2013
Canada	Quebec	Lentic	Rouyn_10	1.62	0.85	0.41	0.36		Ponton and Hare, 2013
Canada	Ontario	Lentic	Kelly_11	3.06	1.62	0.92	0.53		Ponton and Hare, 2013
USA	Delaware	Lotic	Delaware River	0.30	0.18	0.07	0.08		Riedel and Sanders 1998
USA	California	Lotic	CI	16.30	1.10	13.70	1.50	3677	Ryu et al. 2011
USA	California	Pond	I-10	15.40	1.30	12.30	1.80	3612	Ryu et al. 2011
USA	California	Pond	C1-2	11.40	5.30	0.70	5.40	5273	Ryu et al. 2011
USA	California	Pond	C2-3	8.40	3.70	0.30	4.40	7252	Ryu et al. 2011
USA	California	Pond	C7-8	8.30	2.10	0.20	6.00	17617	Ryu et al. 2011
USA	California	Pond	C8-9	12.00	5.70	0.10	6.20	37535	Ryu et al. 2011
USA	California	Pond	C9-10	11.40	6.60	0.90	3.90	41173	Ryu et al. 2011
USA	California	Pond	C10	20.30	11.60	0.60	8.00	51531	Ryu et al. 2011
Canada	Bristish Columbia	Lentic	Goddard Marsh	16.00	2.25	12.00	0.85		Martin et al. 2011
Canada	Bristish Columbia	Lentic	Fording River Oxbow	24.00	0.75	17.50	0.30		Martin et al. 2011
USA	Montana	Lotic	Benton Lake, Stream	98.83	3.07	86.79	8.97		Zhang and Moore, 1996
USA	Montana	Lentic	Benton Lake, Pond	8.14	0.73	4.57	2.83		Zhang and Moore, 1996
Poland	Gniezno	Lentic	Lake 1	0.31	0.28	0.03			Niedzelski, 2006
Poland	Gniezno	Lentic	Lake 2	0.30	0.24	0.06			Niedzelski, 2006
Poland	Gniezno	Lentic	Lake 3	0.30	0.27	0.03			Niedzelski, 2006
Poland	Gniezno	Lentic	Lake 4	0.35	0.22	0.13			Niedzelski, 2006
Poland	Gniezno	Lentic	Lake 8	0.31	0.27	0.04			Niedzelski, 2006
Poland	Gniezno	Lentic	Lake 9	0.18	0.15	0.03			Niedzelski, 2006
Poland	Gniezno	Lentic	Lake 10	0.29	0.26	0.03			Niedzelski, 2006
Poland	Gniezno	Lentic	Lake 11	0.20	0.16	0.04			Niedzelski, 2006
Poland	Gniezno	Lentic	Lake 12	0.25	0.19	0.06			Niedzelski, 2006
Poland	Gniezno	Lentic	Lake 13	0.16	0.12	0.04			Niedzelski, 2006
Poland	Gniezno	Lentic	Lake 14	0.20	0.17	0.03			Niedzelski, 2006
Poland	Gniezno	Lentic	Lake 15	0.20	0.17	0.03			Niedzelski, 2006
USA	West Virginia	Creek	Little Scary	16.50	2.90				Reash 2012
USA	Control	River	Kanawha	NA	NA				Reash 2012
USA	West Virginia	Creek	Conner	129.00	27.00				Reash 2012
USA	West Virginia	Creek	Fish	16.30	10.10				Reash 2012
USA	Kentucky	Creek	Blaine	5.00	0.25				Reash 2012
USA	Indiana	River	Ohio	5.20	0.25				Reash 2012
USA	Indiana	River	Ohio	0.25	0.70				Reash 2012

Table S3: Algae Se concentrations at different aqueous selenite, selenate, and sulphate exposure concentrations

Sample	Se species	SO4 (M)	Water [Se] (M)	Algae [Se] ug/g dw	Reference
Anabaena flos-aquae (cyanobacterium)	Se(IV)	2.8E-05	1.3E-05	271.0	Kiffney and Knight 1990
Anabaena flos-aquae (cyanobacterium)	Se(IV)	2.8E-05	3.7E-05	1071.0	Kiffney and Knight 1990
Anabaena flos-aquae (cyanobacterium)	Se(IV)	2.8E-05	6.3E-05	6072.0	Kiffney and Knight 1990
Anabaena flos-aquae (cyanobacterium)	Se(IV)	2.8E-05	1.3E-04	9365.0	Kiffney and Knight 1990
Chlamydomonas reinhardtii (green alga)	Se(IV)	6.2E-04	1.0E-08	2.1	Besser et al. 1993
Chlamydomonas reinhardtii (green alga)	Se(IV)	6.2E-04	1.1E-07	18.6	Besser et al. 1993
Chlamydomonas reinhardtii (green alga)	Se(IV)	6.2E-04	1.2E-06	46.1	Besser et al. 1993
Scenedesmus obliquus (green alga)	Se(IV)	NA	6.8E-08	1.1	Guan and Wang 2004
Scenedesmus obliquus (green alga)	Se(IV)	NA	6.6E-07	5.3	Guan and Wang 2004
Scenedesmus obliquus (green alga)	Se(IV)	NA	4.7E-06	21.1	Guan and Wang 2004
Scenedesmus obliquus (green alga)	Se(IV)	NA	6.3E-06	105.3	Guan and Wang 2004
Chlamydomonas reinhardtii (green alga)	Se(IV)	NA	1.8E-08	1.1	Guan and Wang 2004
Chlamydomonas reinhardtii (green alga)	Se(IV)	NA	5.3E-08	5.3	Guan and Wang 2004
Chlamydomonas reinhardtii (green alga)	Se(IV)	NA	1.2E-07	21.1	Guan and Wang 2004
Chlamydomonas reinhardtii (green alga)	Se(IV)	NA	3.9E-07	105.3	Guan and Wang 2004
Natural periphyton biofilms	Se(IV)	4.3E-04	3.0E-08	2.0	Conley et al. 2009
Natural periphyton biofilms	Se(IV)	4.3E-04	3.0E-08	2.1	Conley et al. 2009
Natural periphyton biofilms	Se(IV)	4.3E-04	6.2E-08	4.3	Conley et al. 2009
Natural periphyton biofilms	Se(IV)	4.3E-04	1.4E-07	12.3	Conley et al. 2009
Natural periphyton biofilms	Se(IV)	4.3E-04	1.3E-07	9.4	Conley et al. 2009
Natural periphyton biofilms	Se(IV)	4.3E-04	1.6E-07	24.8	Conley et al. 2009
Natural periphyton biofilms	Se(IV)	4.3E-04	1.8E-07	21.5	Conley et al. 2009
Natural periphyton biofilms	Se(IV)	4.3E-04	1.4E-08	4.3	Conley et al. 2011
Natural periphyton biofilms	Se(IV)	4.3E-04	7.5E-08	15.1	Conley et al. 2011
Natural periphyton biofilms	Se(IV)	4.3E-04	2.7E-07	30.9	Conley et al. 2011
Natural periphyton biofilms	Se(IV)	4.3E-04	3.4E-08	11.2	Conley et al. 2011
Natural periphyton biofilms	Se(IV)	4.3E-04	9.0E-08	21.2	Conley et al. 2011
Natural periphyton biofilms	Se(IV)	4.3E-04	2.4E-07	45.3	Conley et al. 2011
Natural periphyton biofilms	Se(IV)	4.3E-04	4.3E-08	10.3	Conley et al. 2011
Natural periphyton biofilms	Se(IV)	4.3E-04	1.1E-07	23.0	Conley et al. 2011
Natural periphyton biofilms	Se(IV)	4.3E-04	2.9E-07	50.7	Conley et al. 2011
Lemna minor (duckweed)	Se(IV)	#VALEUR!	6.3E-09	0.2	Nautilus 2013a
Lemna minor (duckweed)	Se(IV)	#VALEUR!	1.3E-07	3.0	Nautilus 2013a
Lemna minor (duckweed)	Se(IV)	#VALEUR!	2.6E-07	4.8	Nautilus 2013a
Lemna minor (duckweed)	Se(IV)	#VALEUR!	5.2E-07	9.8	Nautilus 2013a
Lemna minor (duckweed)	Se(IV)	1.9E-03	1.6E-07	4.1	Nautilus 2013a
Lemna minor (duckweed)	Se(IV)	#VALEUR!	1.5E-07	3.9	Nautilus 2013a
Lemna minor (duckweed)	Se(IV)	#VALEUR!	1.3E-07	1.9	Nautilus 2013a
Lemna minor (duckweed)	Se(IV)	#VALEUR!	1.3E-07	3.0	Nautilus 2013a
Scenedesmus quadricauda	Se(IV)	4.0E-03	1.3E-04	689.0	Umysova et al. 2009
Scenedesmus quadricauda	Se(IV)	4.0E-02	1.3E-04	678.0	Umysova et al. 2009
Scenedesmus quadricauda	Se(IV)	4.0E-01	1.3E-04	706.0	Umysova et al. 2009
Scenedesmus quadricauda	Se(IV)	4.0E-02	6.3E-04	3730.0	Umysova et al. 2009
Scenedesmus quadricauda	Se(IV)	4.0E-01	6.3E-04	3500.0	Umysova et al. 2009
Scenedesmus quadricauda	Se(IV)	4.0E-03	6.3E-04	1433.0	Vitova et al. 2011
Chlamydomonas reinhardtii	Se(IV)	1.0E-09	5.0E-08	41.3	Morlon et al. 2006
Chlamydomonas reinhardtii	Se(IV)	8.0E-08	5.0E-08	31.8	Morlon et al. 2006
Chlamydomonas reinhardtii	Se(IV)	8.0E-07	5.0E-08	17.9	Morlon et al. 2006
Chlamydomonas reinhardtii	Se(IV)	8.0E-06	5.0E-08	6.5	Morlon et al. 2006
Chlamydomonas reinhardtii	Se(IV)	8.0E-05	5.0E-08	5.9	Morlon et al. 2006
Chlamydomonas reinhardtii	Se(IV)	8.0E-04	5.0E-08	2.0	Morlon et al. 2006
Chlamydomonas reinhardtii	Se(IV)	1.0E-09	5.0E-08	48.0	Morlon et al. 2006
Chlamydomonas reinhardtii	Se(IV)	8.0E-08	5.0E-08	41.3	Morlon et al. 2006
Chlamydomonas reinhardtii	Se(IV)	8.0E-07	5.0E-08	25.3	Morlon et al. 2006
Chlamydomonas reinhardtii	Se(IV)	8.0E-06	5.0E-08	22.1	Morlon et al. 2006
Chlamydomonas reinhardtii	Se(IV)	8.0E-05	5.0E-08	15.4	Morlon et al. 2006
Chlamydomonas reinhardtii	Se(IV)	8.0E-04	5.0E-08	7.4	Morlon et al. 2006
Chlamydomonas reinhardtii	Se(IV)	1.0E-10	6.3E-08	3.9	Ponton et al. 2018
Chlamydomonas reinhardtii	Se(IV)	6.3E-08	6.3E-08	3.3	Ponton et al. 2018
Chlamydomonas reinhardtii	Se(IV)	6.3E-07	6.3E-08	1.6	Ponton et al. 2018
Chlamydomonas reinhardtii	Se(IV)	3.0E-06	6.3E-08	1.3	Ponton et al. 2018
Chlamydomonas reinhardtii	Se(IV)	6.0E-06	6.3E-08	1.4	Ponton et al. 2018
Chlamydomonas reinhardtii	Se(IV)	6.0E-05	6.3E-08	1.3	Ponton et al. 2018

Chlamydomonas reinhardtii	Se(IV)	1.0E-04	6.3E-08	1.3	Ponton et al. 2018
Chlamydomonas reinhardtii	Se(IV)	2.0E-04	6.3E-08	1.2	Ponton et al. 2018
Chlamydomonas reinhardtii	Se(IV)	8.0E-04	6.3E-08	1.3	Ponton et al. 2018
Chlamydomonas reinhardtii	Se(IV)	1.6E-03	6.3E-08	1.1	Ponton et al. 2018
Chrysophyceae, Dynophyceae, Euglenophyceae, bacteria	Se(IV)	1.0E-09	6.3E-08	56.5	Ponton et al. 2018
Chrysophyceae, Dynophyceae, Euglenophyceae, bacteria	Se(IV)	3.0E-06	6.3E-08	55.8	Ponton et al. 2018
Chrysophyceae, Dynophyceae, Euglenophyceae, bacteria	Se(IV)	3.0E-05	6.3E-08	51.2	Ponton et al. 2018
Chrysophyceae, Dynophyceae, Euglenophyceae, bacteria	Se(IV)	2.5E-04	6.3E-08	44.6	Ponton et al. 2018
Chrysophyceae, Dynophyceae, Euglenophyceae, bacteria	Se(IV)	3.0E-03	6.3E-08	47.0	Ponton et al. 2018
Arthrospira platensis	Se(IV)	6.6E-03	1.3E-07	22.0	Li et al. 2003
Arthrospira platensis	Se(IV)	6.6E-03	5.1E-06	219.0	Li et al. 2003
Chlorophyta (natural periphyton)	Se(IV)	3.0E-05	6.3E-08	33.1	Markwart et al. 2018
Bacillariophyta (natural periphyton)	Se(IV)	4.0E-05	6.3E-08	7.3	Markwart et al. 2018
Chlorophyta/Bacillariophyta (natural periphyton)	Se(IV)	6.0E-05	6.3E-08	22.6	Markwart et al. 2018
Chlorophyta	Se(IV)	3.0E-05	6.3E-08	33.7	Markwart et al. 2018
Cyanophyta	Se(IV)	3.0E-06	6.3E-08	119.1	Markwart et al. 2018
Chlorophyta (natural periphyton)	Se(IV)	3.0E-05	3.2E-07	46.9	Markwart et al. 2018
Bacillariophyta (natural periphyton)	Se(IV)	4.0E-05	3.2E-07	16.8	Markwart et al. 2018
Chlorophyta/Bacillariophyta (natural periphyton)	Se(IV)	6.0E-05	3.2E-07	49.7	Markwart et al. 2018
Chlorophyta	Se(IV)	3.0E-05	3.2E-07	65.7	Markwart et al. 2018
Cyanophyta	Se(IV)	3.0E-06	3.2E-07	222.5	Markwart et al. 2018
Chlamydomonas reinhardtii	Se(IV)	4.7E-05	1.3E-07	19.7	Riedel and Sanders 1996
Chlamydomonas reinhardtii	Se(IV)	9.8E-05	1.3E-07	12.1	Riedel and Sanders 1996
Chlamydomonas reinhardtii	Se(IV)	2.0E-04	1.3E-07	10.9	Riedel and Sanders 1996
Chlamydomonas reinhardtii	Se(IV)	5.0E-04	1.3E-07	9.9	Riedel and Sanders 1996
Chlamydomonas reinhardtii	Se(IV)	1.0E-03	1.3E-07	7.1	Riedel and Sanders 1996
Chlamydomonas reinhardtii	Se(IV)	4.7E-05	3.8E-08	9.4	Riedel and Sanders 1996
Chlamydomonas reinhardtii	Se(IV)	9.8E-05	3.8E-08	6.5	Riedel and Sanders 1996
Chlamydomonas reinhardtii	Se(IV)	2.0E-04	3.8E-08	6.9	Riedel and Sanders 1996
Chlamydomonas reinhardtii	Se(IV)	5.0E-04	3.8E-08	5.5	Riedel and Sanders 1996
Chlamydomonas reinhardtii	Se(IV)	1.0E-03	3.8E-08	4.2	Riedel and Sanders 1996
Anabaena flos-aquae (cyanobacterium)	Se(VI)	2.8E-05	1.3E-05	30.4	Kiffney and Knight 1990
Anabaena flos-aquae (cyanobacterium)	Se(VI)	2.8E-05	3.8E-05	744.0	Kiffney and Knight 1990
Anabaena flos-aquae (cyanobacterium)	Se(VI)	2.8E-05	6.2E-05	1218.0	Kiffney and Knight 1990
Anabaena flos-aquae (cyanobacterium)	Se(VI)	2.8E-05	1.2E-04	1313.0	Kiffney and Knight 1990
Chlamydomonas reinhardtii (green alga)	Se(VI)	6.2E-04	1.2E-07	3.7	Besser et al. 1993
Chlamydomonas reinhardtii (green alga)	Se(VI)	6.2E-04	1.2E-06	31.1	Besser et al. 1993
Chlamydomonas reinhardtii (green alga)	Se(VI)	6.2E-04	1.2E-05	367.0	Besser et al. 1993
Chlorella pyrenoidosa (green alga)	Se(VI)	4.0E-05	3.2E-05	3100.0	Bennett et al. 1986
Chlorella pyrenoidosa (green alga)	Se(VI)	4.0E-05	3.2E-05	2600.0	Bennett et al. 1986
Selenastrum capricornutum (green alga)	Se(VI)	1.5E-04	5.1E-08	1.1	Malchow et al. 1995
Selenastrum capricornutum (green alga)	Se(VI)	1.5E-04	1.3E-07	2.1	Malchow et al. 1995
Selenastrum capricornutum (green alga)	Se(VI)	1.5E-04	5.1E-07	7.2	Malchow et al. 1995
Selenastrum capricornutum (green alga)	Se(VI)	3.4E-05	1.4E-07	17.0	Williams et al. 1994
Selenastrum capricornutum (green alga)	Se(VI)	3.4E-05	1.4E-06	156.0	Williams et al. 1994
Selenastrum capricornutum (green alga)	Se(VI)	3.4E-04	1.4E-07	4.0	Williams et al. 1994
Selenastrum capricornutum (green alga)	Se(VI)	3.4E-04	1.4E-06	11.0	Williams et al. 1994
Chlorella vulgaris (green alga)	Se(VI)	1.5E-04	1.3E-06	35.0	Dobbs et al. 1996
Chlorella vulgaris (green alga)	Se(VI)	1.5E-04	2.6E-06	75.0	Dobbs et al. 1996
Chlorella vulgaris (green alga)	Se(VI)	1.5E-04	5.0E-06	155.0	Dobbs et al. 1996
Blue-green algae mixture	Se(VI)	NA	9.2E-08	6.3	Thomas et al. 1999
Blue-green algae mixture	Se(VI)	NA	1.1E-06	36.6	Thomas et al. 1999
Blue-green algae mixture	Se(VI)	NA	1.1E-05	111.0	Thomas et al. 1999
Pseudokirchneriella subcapitata (green alga)	Se(VI)	1.8E-04	5.8E-09	1.2	Rickwood and Jatar 2013
Pseudokirchneriella subcapitata (green alga)	Se(VI)	1.8E-04	1.4E-08	0.7	Rickwood and Jatar 2013
Pseudokirchneriella subcapitata (green alga)	Se(VI)	1.8E-04	1.6E-08	2.2	Rickwood and Jatar 2013
Pseudokirchneriella subcapitata (green alga)	Se(VI)	1.8E-04	2.4E-08	1.2	Rickwood and Jatar 2013
Pseudokirchneriella subcapitata (green alga)	Se(VI)	1.8E-04	3.3E-08	2.9	Rickwood and Jatar 2013
Pseudokirchneriella subcapitata (green alga)	Se(VI)	1.8E-04	3.4E-08	2.1	Rickwood and Jatar 2013
Pseudokirchneriella subcapitata (green alga)	Se(VI)	1.8E-04	4.3E-08	4.3	Rickwood and Jatar 2013
Pseudokirchneriella subcapitata (green alga)	Se(VI)	1.8E-04	6.3E-08	2.8	Rickwood and Jatar 2013
Pseudokirchneriella subcapitata (green alga)	Se(VI)	1.8E-04	8.2E-08	4.0	Rickwood and Jatar 2013
Pseudokirchneriella subcapitata (green alga)	Se(VI)	1.8E-04	9.8E-08	8.0	Rickwood and Jatar 2013
Pseudokirchneriella subcapitata (green alga)	Se(VI)	1.8E-04	1.0E-07	2.3	Rickwood and Jatar 2013
Pseudokirchneriella subcapitata (green alga)	Se(VI)	1.8E-04	1.3E-07	16.6	Rickwood and Jatar 2013
Pseudokirchneriella subcapitata (green alga)	Se(VI)	1.8E-04	1.9E-07	14.6	Rickwood and Jatar 2013

Pseudokirchneriella subcapitata (green alga)	Se(VI)	1.8E-04	2.0E-07	7.8	Rickwood and Jatar 2013
Pseudokirchneriella subcapitata (green alga)	Se(VI)	1.8E-04	2.7E-07	32.4	Rickwood and Jatar 2013
Pseudokirchneriella subcapitata (green alga)	Se(VI)	1.8E-04	3.4E-07	16.2	Rickwood and Jatar 2013
Pseudokirchneriella subcapitata (green alga)	Se(VI)	1.8E-04	3.9E-07	41.6	Rickwood and Jatar 2013
Pseudokirchneriella subcapitata (green alga)	Se(VI)	1.8E-04	5.2E-07	29.3	Rickwood and Jatar 2013
Pseudokirchneriella subcapitata (green alga)	Se(VI)	1.8E-04	5.2E-07	54.2	Rickwood and Jatar 2013
Pseudokirchneriella subcapitata (green alga)	Se(VI)	1.8E-04	5.2E-07	33.3	Rickwood and Jatar 2013
Pseudokirchneriella subcapitata (green alga)	Se(VI)	1.8E-04	5.5E-07	24.9	Rickwood and Jatar 2013
Pseudokirchneriella subcapitata (green alga)	Se(VI)	1.8E-04	5.5E-07	37.5	Rickwood and Jatar 2013
Pseudokirchneriella subcapitata (green alga)	Se(VI)	1.8E-04	6.4E-07	20.2	Rickwood and Jatar 2013
Pseudokirchneriella subcapitata (green alga)	Se(VI)	1.8E-04	6.6E-07	46.6	Rickwood and Jatar 2013
Pseudokirchneriella subcapitata (green alga)	Se(VI)	1.8E-04	6.6E-07	25.3	Rickwood and Jatar 2013
Pseudokirchneriella subcapitata (green alga)	Se(VI)	1.8E-04	6.6E-07	28.1	Rickwood and Jatar 2013
Pseudokirchneriella subcapitata (green alga)	Se(VI)	1.8E-04	7.6E-07	184.4	Rickwood and Jatar 2013
Pseudokirchneriella subcapitata (green alga)	Se(VI)	1.8E-04	1.3E-06	98.5	Rickwood and Jatar 2013
Pseudokirchneriella subcapitata (green alga)	Se(VI)	1.8E-04	1.3E-06	91.6	Rickwood and Jatar 2013
Pseudokirchneriella subcapitata (green alga)	Se(VI)	1.8E-04	1.4E-06	96.3	Rickwood and Jatar 2013
Pseudokirchneriella subcapitata (green alga)	Se(VI)	1.8E-04	1.6E-06	314.3	Rickwood and Jatar 2013
Chlorella vulgaris (green alga)	Se(VI)	1.8E-04	6.3E-09	1.4	Rickwood and Jatar 2013
Chlorella vulgaris (green alga)	Se(VI)	1.8E-04	1.4E-08	2.3	Rickwood and Jatar 2013
Chlorella vulgaris (green alga)	Se(VI)	1.8E-04	2.0E-08	0.2	Rickwood and Jatar 2013
Chlorella vulgaris (green alga)	Se(VI)	1.8E-04	3.5E-08	7.5	Rickwood and Jatar 2013
Chlorella vulgaris (green alga)	Se(VI)	1.8E-04	5.1E-08	0.5	Rickwood and Jatar 2013
Chlorella vulgaris (green alga)	Se(VI)	1.8E-04	7.7E-08	5.5	Rickwood and Jatar 2013
Chlorella vulgaris (green alga)	Se(VI)	1.8E-04	8.5E-08	4.4	Rickwood and Jatar 2013
Chlorella vulgaris (green alga)	Se(VI)	1.8E-04	9.1E-08	1.5	Rickwood and Jatar 2013
Chlorella vulgaris (green alga)	Se(VI)	1.8E-04	1.2E-07	1.2	Rickwood and Jatar 2013
Chlorella vulgaris (green alga)	Se(VI)	1.8E-04	1.3E-07	7.4	Rickwood and Jatar 2013
Chlorella vulgaris (green alga)	Se(VI)	1.8E-04	1.5E-07	2.6	Rickwood and Jatar 2013
Chlorella vulgaris (green alga)	Se(VI)	1.8E-04	2.1E-07	6.9	Rickwood and Jatar 2013
Chlorella vulgaris (green alga)	Se(VI)	1.8E-04	2.5E-07	14.4	Rickwood and Jatar 2013
Chlorella vulgaris (green alga)	Se(VI)	1.8E-04	2.8E-07	24.5	Rickwood and Jatar 2013
Chlorella vulgaris (green alga)	Se(VI)	1.8E-04	3.1E-07	5.0	Rickwood and Jatar 2013
Chlorella vulgaris (green alga)	Se(VI)	1.8E-04	4.9E-07	39.1	Rickwood and Jatar 2013
Chlorella vulgaris (green alga)	Se(VI)	1.8E-04	5.0E-07	27.0	Rickwood and Jatar 2013
Chlorella vulgaris (green alga)	Se(VI)	1.8E-04	5.2E-07	14.0	Rickwood and Jatar 2013
Chlorella vulgaris (green alga)	Se(VI)	1.8E-04	5.3E-07	35.7	Rickwood and Jatar 2013
Chlorella vulgaris (green alga)	Se(VI)	1.8E-04	5.4E-07	13.0	Rickwood and Jatar 2013
Chlorella vulgaris (green alga)	Se(VI)	1.8E-04	5.9E-07	7.9	Rickwood and Jatar 2013
Chlorella vulgaris (green alga)	Se(VI)	1.8E-04	5.9E-07	9.5	Rickwood and Jatar 2013
Chlorella vulgaris (green alga)	Se(VI)	1.8E-04	5.9E-07	10.6	Rickwood and Jatar 2013
Chlorella vulgaris (green alga)	Se(VI)	1.8E-04	6.0E-07	22.1	Rickwood and Jatar 2013
Chlorella vulgaris (green alga)	Se(VI)	1.8E-04	1.2E-06	159.4	Rickwood and Jatar 2013
Chlorella vulgaris (green alga)	Se(VI)	1.8E-04	1.2E-06	44.7	Rickwood and Jatar 2013
Chlorella vulgaris (green alga)	Se(VI)	1.8E-04	1.3E-06	68.3	Rickwood and Jatar 2013
Chlorella vulgaris (green alga)	Se(VI)	1.8E-04	1.3E-06	52.9	Rickwood and Jatar 2013
Chlorella vulgaris (green alga)	Se(VI)	1.8E-04	1.4E-06	78.5	Rickwood and Jatar 2013
Chlamydomonas reinhardtii	Se(VI)	8.0E-06	1.0E-08	16.4	Fournier et al. 2010
Chlamydomonas reinhardtii	Se(VI)	8.0E-06	1.0E-07	56.5	Fournier et al. 2010
Chlamydomonas reinhardtii	Se(VI)	8.0E-06	6.0E-07	889.2	Fournier et al. 2010
Chlamydomonas reinhardtii	Se(VI)	8.0E-06	2.5E-06	1012.0	Fournier et al. 2010
Chlamydomonas reinhardtii	Se(VI)	8.0E-05	1.0E-08	1.5	Fournier et al. 2010
Chlamydomonas reinhardtii	Se(VI)	8.0E-05	1.0E-07	6.0	Fournier et al. 2010
Chlamydomonas reinhardtii	Se(VI)	8.0E-05	6.0E-07	31.0	Fournier et al. 2010
Chlamydomonas reinhardtii	Se(VI)	8.0E-05	2.5E-06	123.8	Fournier et al. 2010
Chlamydomonas reinhardtii	Se(VI)	8.0E-05	2.5E-06	131.5	Geoffroy et al. 2007
Chlamydomonas reinhardtii	Se(VI)	8.0E-05	5.0E-06	345.9	Geoffroy et al. 2007
Chlamydomonas reinhardtii	Se(VI)	8.0E-05	7.5E-06	567.5	Geoffroy et al. 2007
Chlamydomonas reinhardtii	Se(VI)	1.0E-09	6.3E-08	224.9	Ponton et al. 2018
Chlamydomonas reinhardtii	Se(VI)	6.3E-08	6.3E-08	215.6	Ponton et al. 2018
Chlamydomonas reinhardtii	Se(VI)	5.0E-07	6.3E-08	154.7	Ponton et al. 2018
Chlamydomonas reinhardtii	Se(VI)	5.0E-06	6.3E-08	28.6	Ponton et al. 2018
Chlamydomonas reinhardtii	Se(VI)	2.5E-05	6.3E-08	4.8	Ponton et al. 2018
Chlamydomonas reinhardtii	Se(VI)	5.0E-05	6.3E-08	1.5	Ponton et al. 2018
Chlamydomonas reinhardtii	Se(VI)	1.0E-04	6.3E-08	1.4	Ponton et al. 2018
Chlamydomonas reinhardtii	Se(VI)	5.0E-04	6.3E-08	0.5	Ponton et al. 2018

Chlamydomonas reinhardtii	Se(VI)	2.0E-03	6.3E-08	0.2	Ponton et al. 2018
Chlamydomonas reinhardtii	Se(VI)	4.0E-03	6.3E-08	0.2	Ponton et al. 2018
Chlamydomonas reinhardtii	Se(VI)	1.5E-04	7.5E-05	837.1	Vriens et al. 2018
Chlamydomonas reinhardtii	Se(VI)	3.0E-04	7.5E-05	567.6	Vriens et al. 2018
Chlamydomonas reinhardtii	Se(VI)	7.5E-04	7.5E-05	384.6	Vriens et al. 2018
Chrysophyceae, Dynophyceae, Euglenophyceae, bacteria	Se(VI)	1.0E-08	6.3E-08	36.5	Ponton et al. 2018
Chrysophyceae, Dynophyceae, Euglenophyceae, bacteria	Se(VI)	6.3E-08	6.3E-08	17.2	Ponton et al. 2018
Chrysophyceae, Dynophyceae, Euglenophyceae, bacteria	Se(VI)	2.2E-06	6.3E-08	13.3	Ponton et al. 2018
Chrysophyceae, Dynophyceae, Euglenophyceae, bacteria	Se(VI)	2.3E-05	6.3E-08	14.0	Ponton et al. 2018
Chrysophyceae, Dynophyceae, Euglenophyceae, bacteria	Se(VI)	3.0E-04	6.3E-08	7.1	Ponton et al. 2018
Chrysophyceae, Dynophyceae, Euglenophyceae, bacteria	Se(VI)	3.0E-03	6.3E-08	5.7	Ponton et al. 2018
Chlorophyta (natural periphyton)	Se(VI)	3.0E-05	6.3E-08	2.7	Markwart et al. 2018
Bacillariophyta (natural periphyton)	Se(VI)	4.0E-05	6.3E-08	3.2	Markwart et al. 2018
Chlorophyta/Bacillariophyta (natural periphyton)	Se(VI)	6.0E-05	6.3E-08	2.2	Markwart et al. 2018
Chlorophyta	Se(VI)	3.0E-05	6.3E-08	3.2	Markwart et al. 2018
Cyanophyta	Se(VI)	3.0E-06	6.3E-08	2.8	Markwart et al. 2018
Chlorophyta (natural periphyton)	Se(VI)	3.0E-05	3.2E-07	7.5	Markwart et al. 2018
Bacillariophyta (natural periphyton)	Se(VI)	4.0E-05	3.2E-07	8.6	Markwart et al. 2018
Chlorophyta/Bacillariophyta (natural periphyton)	Se(VI)	6.0E-05	3.2E-07	6.9	Markwart et al. 2018
Chlorophyta	Se(VI)	3.0E-05	3.2E-07	9.5	Markwart et al. 2018
Cyanophyta	Se(VI)	3.0E-06	3.2E-07	3.9	Markwart et al. 2018
Pseudokirchneriella subcapitata (green alga)	Se(VI)	5.2E-05	1.3E-07	2.5	Lo et al. 2015
Pseudokirchneriella subcapitata (green alga)	Se(VI)	5.2E-05	2.3E-07	3.4	Lo et al. 2015
Pseudokirchneriella subcapitata (green alga)	Se(VI)	5.2E-05	3.8E-07	7.1	Lo et al. 2015
Pseudokirchneriella subcapitata (green alga)	Se(VI)	5.2E-05	6.8E-07	13.3	Lo et al. 2015
Pseudokirchneriella subcapitata (green alga)	Se(VI)	5.2E-05	1.2E-06	20.4	Lo et al. 2015
Pseudokirchneriella subcapitata (green alga)	Se(VI)	1.6E-03	1.1E-07	1.0	Lo et al. 2015
Pseudokirchneriella subcapitata (green alga)	Se(VI)	1.6E-03	2.3E-07	1.2	Lo et al. 2015
Pseudokirchneriella subcapitata (green alga)	Se(VI)	1.6E-03	3.9E-07	2.1	Lo et al. 2015
Pseudokirchneriella subcapitata (green alga)	Se(VI)	1.6E-03	7.1E-07	2.8	Lo et al. 2015
Pseudokirchneriella subcapitata (green alga)	Se(VI)	1.6E-03	1.2E-06	4.6	Lo et al. 2015
Pseudokirchneriella subcapitata (green alga)	Se(VI)	4.1E-03	1.3E-07	0.3	Lo et al. 2015
Pseudokirchneriella subcapitata (green alga)	Se(VI)	4.1E-03	2.3E-07	0.3	Lo et al. 2015
Pseudokirchneriella subcapitata (green alga)	Se(VI)	4.1E-03	3.9E-07	0.7	Lo et al. 2015
Pseudokirchneriella subcapitata (green alga)	Se(VI)	4.1E-03	7.0E-07	1.8	Lo et al. 2015
Pseudokirchneriella subcapitata (green alga)	Se(VI)	4.1E-03	1.3E-06	1.1	Lo et al. 2015
Lemna minor	Se(VI)	5.3E-04	5.7E-08	1.4	Lo et al. 2015
Lemna minor	Se(VI)	5.3E-04	1.1E-07	2.6	Lo et al. 2015
Lemna minor	Se(VI)	5.3E-04	2.3E-07	5.5	Lo et al. 2015
Lemna minor	Se(VI)	5.3E-04	4.7E-07	10.3	Lo et al. 2015
Lemna minor	Se(VI)	1.4E-03	4.8E-08	0.6	Lo et al. 2015
Lemna minor	Se(VI)	1.4E-03	1.2E-07	1.1	Lo et al. 2015
Lemna minor	Se(VI)	1.4E-03	2.5E-07	2.3	Lo et al. 2015
Lemna minor	Se(VI)	1.4E-03	4.9E-07	3.8	Lo et al. 2015
Lemna minor	Se(VI)	2.3E-03	5.3E-08	0.5	Lo et al. 2015
Lemna minor	Se(VI)	2.3E-03	1.2E-07	1.4	Lo et al. 2015
Lemna minor	Se(VI)	2.3E-03	2.3E-07	2.0	Lo et al. 2015
Lemna minor	Se(VI)	2.3E-03	5.0E-07	3.4	Lo et al. 2015
Lemna minor	Se(VI)	3.5E-03	6.0E-08	0.3	Lo et al. 2015
Lemna minor	Se(VI)	3.5E-03	1.2E-07	0.7	Lo et al. 2015
Lemna minor	Se(VI)	3.5E-03	2.5E-07	1.1	Lo et al. 2015
Lemna minor	Se(VI)	3.5E-03	4.9E-07	1.9	Lo et al. 2015
Ulva	Se(VI)	3.1E-02	2.5E-06	0.6	Schiavon et al. 2012
Ulva	Se(VI)	3.1E-02	1.0E-05	1.7	Schiavon et al. 2012
Ulva	Se(VI)	3.1E-02	5.0E-05	13.3	Schiavon et al. 2012
Ulva	Se(VI)	3.1E-02	7.5E-05	21.3	Schiavon et al. 2012
Ulva	Se(VI)	3.1E-02	1.0E-04	30.8	Schiavon et al. 2012
Selenastrum capricornutum	Se(VI)	3.4E-05	1.3E-07	17.0	William et al. 1991
Selenastrum capricornutum	Se(VI)	3.4E-05	1.3E-06	156.0	William et al. 1991
Selenastrum capricornutum	Se(VI)	3.4E-04	1.3E-07	4.0	William et al. 1991
Selenastrum capricornutum	Se(VI)	3.4E-04	1.3E-06	11.0	William et al. 1991
